# Proteomic quantification of native and ECM-enriched mouse ovaries reveals an age-dependent fibro-inflammatory signature

**DOI:** 10.18632/aging.205190

**Published:** 2023-10-27

**Authors:** Shweta S. Dipali, Christina D. King, Jacob P. Rose, Joanna E. Burdette, Judith Campisi, Birgit Schilling, Francesca E. Duncan

**Affiliations:** 1 Department of Obstetrics and Gynecology, Feinberg School of Medicine, Northwestern University, Chicago, IL 60611, USA; 2 Buck Institute for Research on Aging, Novato, CA 94945, USA; 3 Department of Pharmaceutical Sciences, University of Illinois at Chicago, Chicago, IL 60607, USA

**Keywords:** reproductive aging, ovary, proteomics, extracellular matrix, data-independent acquisition

## Abstract

The ovarian microenvironment becomes fibrotic and stiff with age, in part due to increased collagen and decreased hyaluronan. However, the extracellular matrix (ECM) is a complex network of hundreds of proteins, glycoproteins, and glycans which are highly tissue specific and undergo pronounced changes with age. To obtain an unbiased and comprehensive profile of age-associated alterations to the murine ovarian proteome and ECM, we used a label-free quantitative proteomic methodology. We validated conditions to enrich for the ECM prior to proteomic analysis. Following analysis by data-independent acquisition (DIA) and quantitative data processing, we observed that both native and ECM-enriched ovaries clustered separately based on age, indicating distinct age-dependent proteomic signatures. We identified a total of 4,721 proteins from both native and ECM-enriched ovaries, of which 383 proteins were significantly altered with advanced age, including 58 ECM proteins. Several ECM proteins upregulated with age have been associated with fibrosis in other organs, but to date their roles in ovarian fibrosis are unknown. Pathways regulating DNA metabolism and translation were downregulated with age, whereas pathways involved in ECM remodeling and immune response were upregulated. Interestingly, immune-related pathways were upregulated with age even in ECM-enriched ovaries, suggesting a novel interplay between the ECM and the immune system. Moreover, we identified putative markers of unique immune cell populations present in the ovary with age. These findings provide evidence from a proteomic perspective that the aging ovary provides a fibroinflammatory milieu, and our study suggests target proteins which may drive these age-associated phenotypes for future investigation.

## INTRODUCTION

Aging is associated with cell and tissue deterioration that contributes to impaired organ function [1]. The ovary is unique in that it is the first organ to exhibit overt signs of aging in the human body [2, 3]. Female fertility begins to decline when women reach their mid-thirties and reproductive function ceases completely at the time of menopause [4]. In the ovary, reproductive aging is characterized by decreased gamete quantity and quality, which ultimately results in adverse fertility, endocrine, and overall health outcomes [5, 6].

The mechanisms underlying ovarian aging are multi-factorial. Age-associated changes intrinsic to the gamete are well characterized and include spindle abnormalities, chromosome missegregation, epigenetic dysregulation, impaired proteostasis, as well as mitochondrial dysfunction [7–9]. However, the oocyte does not exist in isolation, but rather is dependent on a dynamic and complex microenvironment. Oocytes are surrounded by companion somatic cells within functional units known as follicles. Ovarian follicles exhibit altered gene expression and endocrine output with advanced reproductive age [10, 11]. Follicle growth and functional maturation are supported by the stromal sub-compartment, composed of fibroblast-interstitial cells, immune cells, blood and lymphatic vessels, nerves, as well as a highly structured extracellular matrix (ECM) [12]. Reproductive aging is associated with a shift to a pro-inflammatory milieu in the ovarian stroma with increased expression of pro-inflammatory cytokines and inflammasome genes [13, 14]. Current reports are contradictory as to how aging alters specific ovarian immune cell populations, but this is likely because the immune environment of the ovary is highly dependent on variables such as age, strain, and estrous cycle stage [13–17]. Broadly, it is thought that ovarian aging results in a larger population of adaptive immune cells, including lymphoid cells, in the ovary [14, 15]. Moreover, reproductive aging is associated with a depletion of tissue-resident macrophages and predominance of monocyte-derived macrophages, a shift in macrophage polarization to favor M2 alternative activation, as well as the presence of a unique population of multinucleated macrophage giant cells (MNGCs) [13, 17, 18].

Inflammation in the ovary is accompanied by increased fibrosis and tissue stiffness, in part due to age-associated alterations to the ECM [13, 16, 19–22]. Specifically, hyaluronan (HA) decreases with age in the ovarian stroma, whereas collagens I and III increase, and importantly, these age-dependent changes are conserved in mouse and human [13, 19]. The ovarian ECM, however, is extensive, containing fibril- and network-forming proteins, proteoglycans, and glycosaminoglycans, including but not limited to several types of collagens, as well as fibronectin and laminins [12, 23–25]. Previous studies which defined age-associated changes to the ovarian ECM using human tissue, identified differentially expressed matrix proteins across the lifespan [26]. These stage-specific ECM proteins may serve as biomarkers of ovarian function as they regulated pathways involved in ECM remodeling and follicle quiescence in the pre-pubertal period, pathways supporting follicle development and angiogenesis during reproductive age, as well as ECM production, fibrosis, and senescence at menopause [26]. However, because human samples are difficult to obtain for research purposes and are limited by participant heterogeneity, we sought to characterize age-related alterations to the ovarian ECM in a tightly controlled murine model of physiologic aging.

The overarching goal of this study was to use label-free quantitative proteomic methods to define comprehensive, age-dependent changes in the murine ovarian proteome and ECM in an unbiased manner. To this end, we first optimized and validated organ-specific methods to enrich for the ECM of mouse ovaries. We then performed proteomic analysis of native and ECM-enriched ovaries from reproductively young and old mice using mass spectrometric data-independent acquisitions (DIA-MS) [27–29]. Our findings demonstrate that ovarian aging generates a fibroinflammatory milieu and lay the foundation for further understanding how the ECM may contribute to aging. Additionally, we identified a series of specific protein targets that may drive reproductive aging phenotypes and be modulated to improve reproductive longevity.

## RESULTS

### Detergent treatment effectively enriches the mouse ovarian ECM

**Figure 1:**
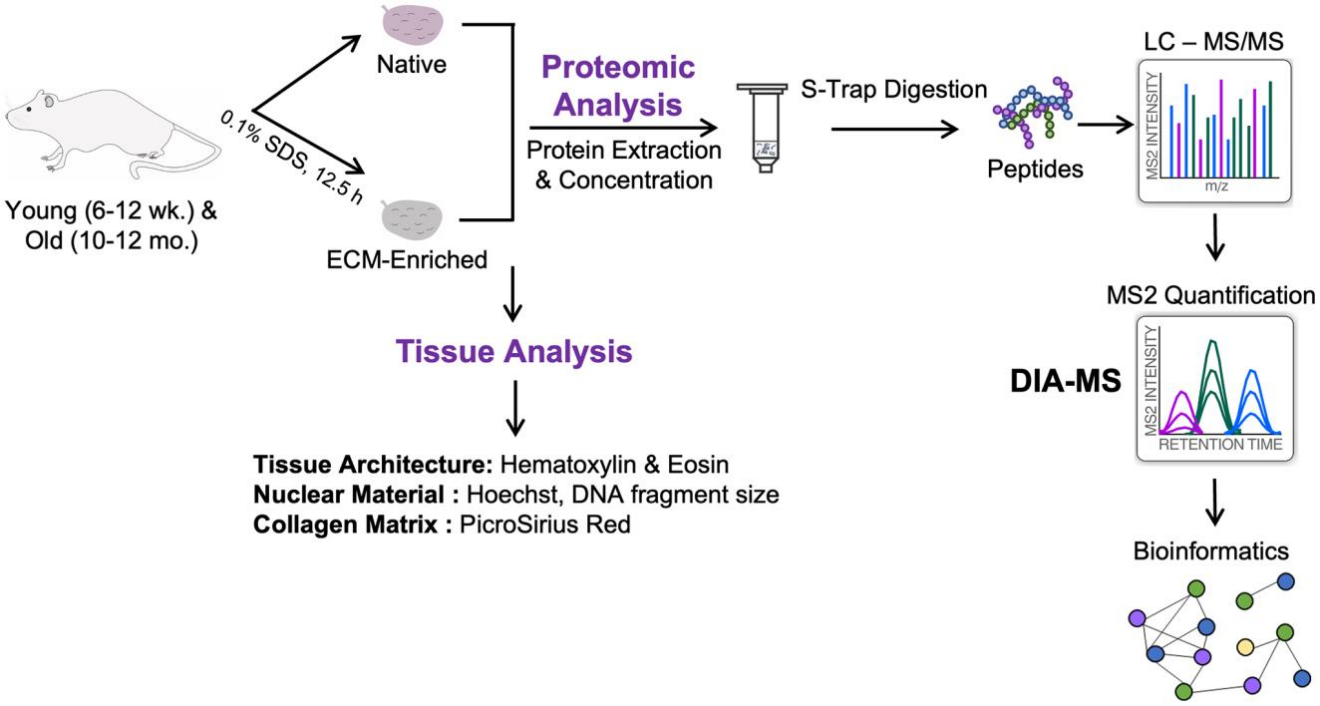
**Schematic of workflow.** Ovaries were harvested from reproductively young (6–12 weeks) and reproductively old (10–12 months) mice. One ovary per mouse was kept native and the contralateral ovary was enriched for the ECM by treatment with 0.1% SDS for 12.5 h. Native and ECM-enriched ovaries were characterized by H&E, Hoechst, and PSR staining of tissue sections, as well as measurement of DNA fragment size on an agarose gel. Tissue was homogenized and proteins were extracted, digested using S-Trap, and desalted by HLB C_18_ cartridges. Peptides were analyzed on an Orbitrap Exploris 480 by Data-Independent Acquisition (DIA)-MS to quantify dynamic protein changes across reproductive aging. Raw data were analyzed using Spectronaut and significantly altered proteins were subjected to pathway analysis using ConsensusPathDB-mouse.

To investigate age-associated changes to the ovarian proteome, we isolated ovaries from reproductively young (6–12 weeks) and old mice (10–12 months) and analyzed them both in their native state and enriched for the ECM to specifically determine how aging alters the ovarian matrix (Figure 1). We, therefore, first optimized an ECM enrichment strategy using a decellularization approach, which removes cellular and nuclear material while preserving the composition and structure of the ECM [30, 31]. Decellularized scaffolds are often used for bioengineering applications, as they can be re-seeded with allogenic cells, which has the benefit of preventing an immune response while maintaining tissue-specific cell phenotypes and functions [32, 33]. We further optimized this technique to enrich for the ECM of mouse ovaries prior to proteomic analysis. We first tested several detergents previously used for decellularizing murine, bovine, porcine, and human ovaries, including 0.5% sodium dodecyl sulfate (SDS), 0.1% SDS, and 1% Triton-X [34–37]. Decellularization has previously been performed in mouse ovaries with the goal of use in bioengineering applications, and as the decellularized tissue had to be completely devoid of cells, this inherently created the tradeoff of compromised ECM integrity [36, 38]. In contrast, our goal was to preserve as much of the mouse ovarian ECM as possible to allow for a comprehensive analysis of age-dependent alterations to the matrix. In our experimental setup, treatment with 1% Triton-X for 24 hours did not result in decellularization, whereas treatment with both 0.5% SDS for 10 hours and 0.1% SDS for 24 hours effectively removed cellular content as evidenced by lack of nuclei in Hematoxylin and Eosin (H&E) stained tissue sections (Supplementary Figure 1). We evaluated matrix integrity using the histological stain PicroSirius Red (PSR) which detects collagen I and III fibers. Compared to 0.5% SDS, treatment with 0.1% SDS appeared to better preserve the integrity of the collagen-matrix (Supplementary Figure 1). Therefore, we further refined the ECM enrichment protocol using 0.1% SDS.

**Figure 2:**
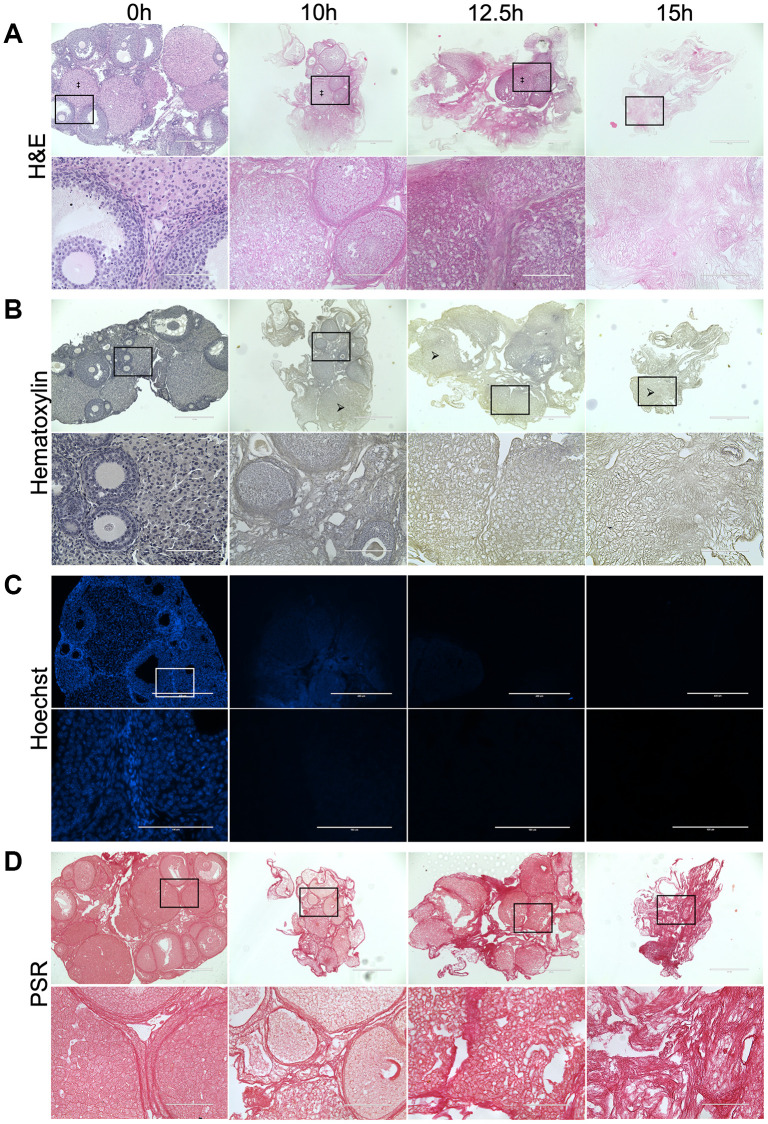
**Treatment with 0.1% SDS enriches for the ovarian ECM in 12.5 hours.** Representative images of H&E (**A**), Hematoxylin (**B**), Hoechst (**C**), and PSR (**D**) stained ovarian tissue sections following treatment with 0.1% SDS for 0 h (native), 10 h, 12.5 h, or 15 h. Bottom row of each panel is optical zoom of boxed region from top row. Scale bar for top row for each panel = 400 μm. Scale bar for bottom row for each panel = 100 μm. *N* = 3–4 ovaries per group. Example of a corpus luteum indicated by ‡ in H&E-stained images. Example of region without nuclei indicated by arrows in Hematoxylin-stained images.

As longer exposure to detergent may compromise the ECM, we performed a time-course with 0.1% SDS treatment for 0, 10, 12.5, or 15 hours to establish the minimum amount of time needed to remove cells without perturbing ECM integrity. Although nuclear material was still visible following 10-hour treatment, most was effectively removed at 12.5 and 15 hours as assessed by H&E, Hematoxylin, and Hoechst staining of tissue sections to visualize DNA (Figure 2A–2C). However, the structure of the collagen-matrix as assessed by PSR staining was better maintained following 0.1% SDS treatment for 12.5 hours compared to 15 hours (Figure 2D). In fact, ovarian structures, including corpora lutea, were still distinguishable in tissue sections from ovaries treated with 0.1% SDS for 12.5 hours (Figure 2A). Thus, we used 0.1% SDS for 12.5 hours to enrich for the ovarian ECM for all downstream studies.

### ECM-enrichment was effective in ovaries from reproductively young and old mice

**Figure 3:**
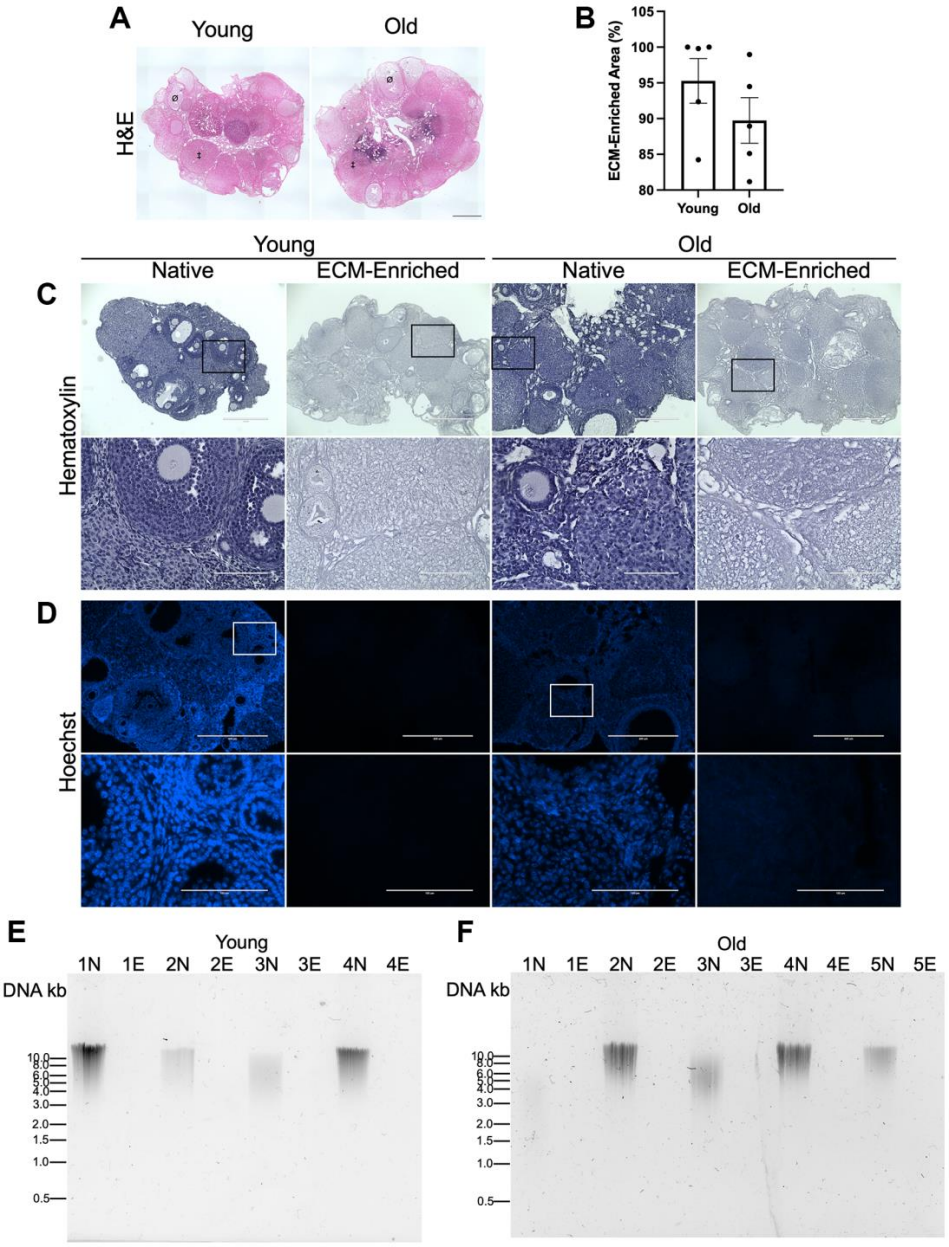
**ECM-enrichment was effective in ovaries from reproductively young and reproductively old mice.** (**A**) Representative scans of ovarian tissue sections from reproductively young and old mice stained with H&E following treatment with 0.1% SDS for 12.5 h. Example of a follicle indicated by Ø and example of a corpus luteum indicated by ‡. (**B**) Quantification of relative ECM-enriched area (%). (**C**, **D**) Representative images of Hematoxylin (**C**) and Hoechst (**D**) stained ovarian tissue sections from reproductively young and old mice following treatment with 0.1% SDS for 0 h (Native) or 12.5 h (ECM-enriched). Bottom row of each panel is optical zoom of boxed region from top row. Scale bar for top row for each panel = 400 μm. Scale bar for bottom row for each panel = 100 μm. (**E**, **F**) Images of agarose gel electrophoresis of DNA extracted from native (N) and ECM-enriched (E) ovaries from reproductively young (**E**) and old (**F**) mice. Numbers (ex: 1N and 1E) correspond to ovaries from the same mouse. Samples run with 1 kb DNA ladder. *N* = 4–5 ovaries per group.

**Figure 4:**
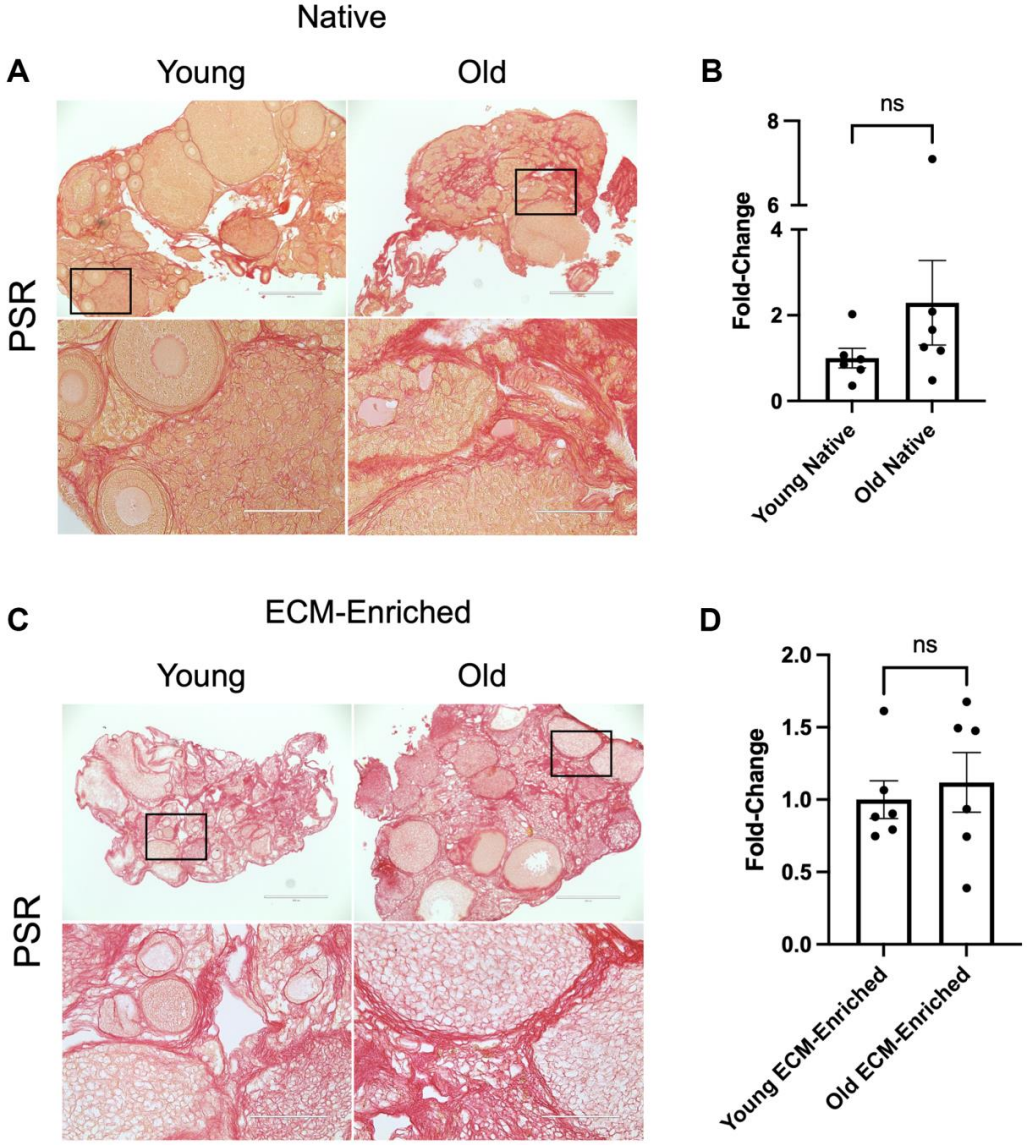
**ECM-enrichment maintains collagen I and III.** (**A**, **C**) Representative images of PSR stained ovarian tissue sections from reproductively young and old mice following treatment with 0.1% SDS for 0 h (**A**, Native) or 12.5 h (**C**, ECM-enriched). Bottom row of each panel is optical zoom of boxed region from top row. Scale bar for top row for each panel = 400 μm. Scale bar for bottom row for each panel = 100 μm. (**B**, **D**) Quantification of fold-change of relative PSR-positive ovarian area in native (**B**) and ECM-enriched (**D**) ovaries from reproductively old mice over young counterparts. *N* = 6 ovaries per group.

To validate that our optimized ECM enrichment strategy was equivalently effective irrespective of animal age, we incubated ovaries from reproductively young and old mice in 0.1% SDS for 12.5 hours in parallel and evaluated ovarian architecture and cellular content through assessment of DNA using various approaches. These conditions maintained the overall structure of the ovaries with regions previously occupied by follicles, corpora lutea, and stroma still clearly visible (Figure 3A). The majority of the H&E-stained tissue lacked visible nuclei, with 95.3 ± 3.1% and 89.7 ± 3.2% of the ovaries from reproductively young and old mice, respectively, being enriched in decellularized matrix (Figure 3A, 3B). We further confirmed these findings by demonstrating the absence of nuclear material in tissue sections from ECM-enriched ovaries relative to native ovaries from reproductively young and old mice stained with Hematoxylin only or Hoechst to detect DNA (Figure 3C, 3D). Moreover, we validated these histological findings by running DNA extracted from native and ECM-enriched ovaries on agarose gels. Whereas DNA bands were clearly detectable in native ovaries, this was not the case in detergent-treated samples irrespective of animal age, further demonstrating effective removal of cellular contents (Figure 3E, 3F). We optimized conditions such that a small percentage of residual nuclei in addition to identifiable ovarian structures were maintained in ECM-enriched tissues, thus maximizing the likelihood that the detergent treatment did not completely strip the tissue to the point of perturbing ECM integrity. Our detergent treatment approach also maintained the integrity of the ovarian ECM as evidenced by positive collagen I and III signal in ECM-enriched ovaries from both reproductively young and old mice, which was similar to that observed in native ovaries (Figure 4). With advanced reproductive age, there is a reported accumulation of collagen I and III in the ovary which ultimately contributes to tissue fibrosis [13, 19, 20]. Although the reproductively old mice used in this study are not as old as those in previous studies where significant age-associated fibrosis was observed, we did see a similar trend of increased collagen in our samples. For example, there was a 2.3 ± 1.0-fold increase in PSR-positive area in native ovaries from reproductively old mice compared to young counterparts (Figure 4A, 4B). In the ECM enriched samples, there was a 1.1 ± 0.2-fold increase in PSR-positive area with age (Figure 4C, 4D). Thus, our ECM enrichment strategy preserved key matrix components and architecture.

### Native and ECM-enriched ovaries have distinct age-associated proteomic signatures

To determine how reproductive aging impacts the ovarian proteome and ECM, we used an unbiased proteomic approach using data-independent acquisition on an Orbitrap Exploris 480 and a direct-DIA method in which the DIA-MS data were used to simultaneously identify and quantify all proteins (Figure 1). For each of the 5 biological replicates per age group, one ovary from each mouse was analyzed in its native state, while the contralateral ovary was enriched for the ECM. Using this experimental approach, we probed age-dependent changes to the whole ovarian proteome and ECM in parallel (Figure 1). For the label-free quantitative proteomic workflow, we lysed the native tissue with 2% SDS and resolubilized the ECM with 2% SDS and 50 mM TEAB, respectively. Samples were then proteolytically digested with trypsin using S-Trap micro spin columns. Following DIA-MS analysis, all data were searched and statistically processed by Spectronaut v14, and subjected to additional pathway analysis and data mining (Figure 1).

**Figure 5:**
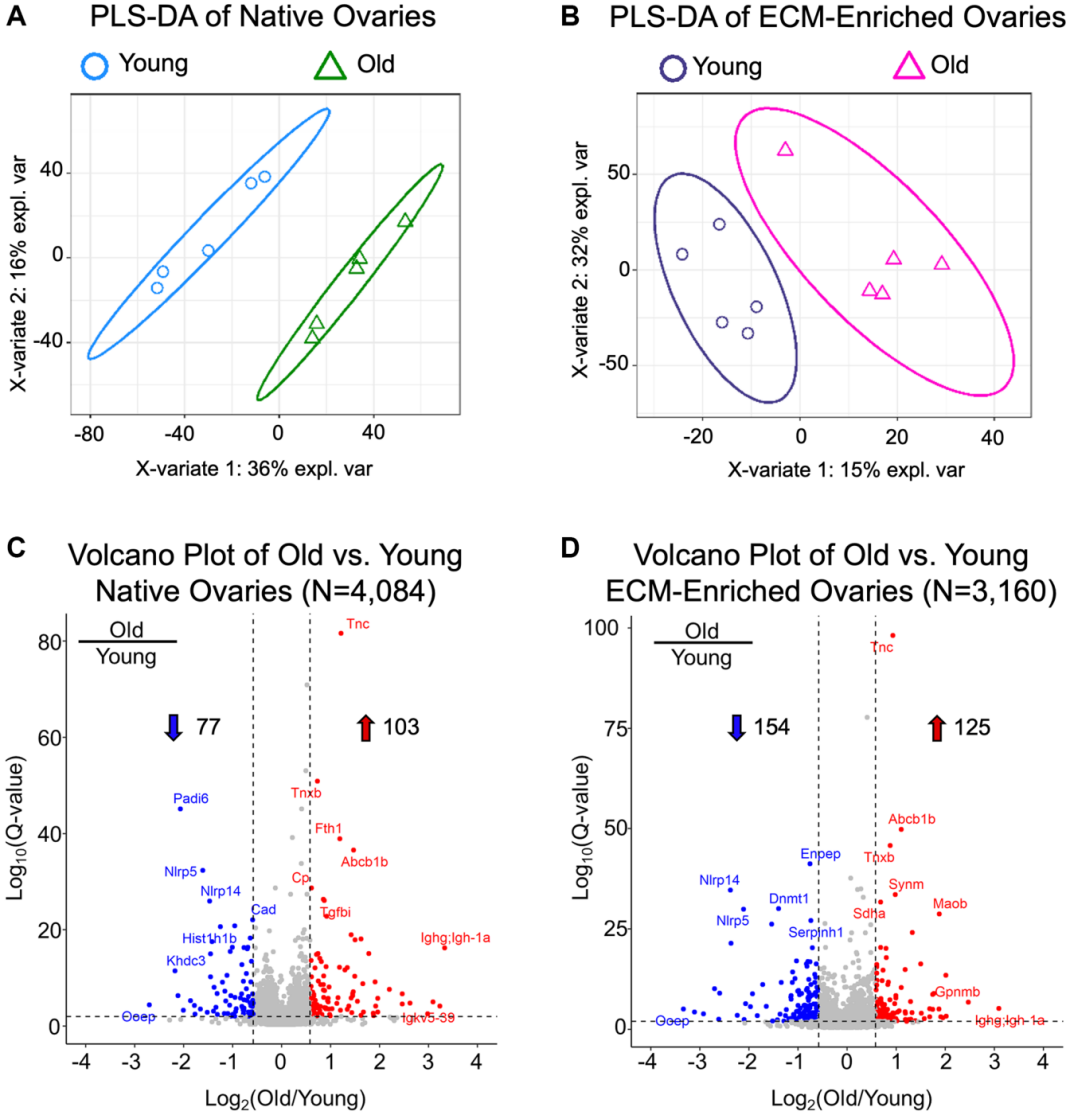
**Native and ECM-enriched ovaries have distinct age-associated proteomic signatures.** (**A**, **B**) Partial least squares-discriminant analysis performed with quantifiable proteins from native (**A**, *N* = 4,084) and ECM-enriched (**B**, *N* = 3,160) ovaries shows distinct, age-dependent clustering in both native and ECM-enriched conditions. (**C**, **D**) Volcano plots of significantly altered proteins comparing (**C**) old vs. young native ovaries (*N* = 180) and (**D**) old vs. young ECM-enriched ovaries (*N* = 279). In native ovaries, 103 proteins are significantly upregulated and 77 proteins are significantly downregulated with age. In ECM-enriched ovaries, 125 proteins are significantly upregulated and 154 proteins are significantly downregulated with age. Filtering criteria to determine significantly altered proteins are as follows: *q*-value < 0.01 and |log_2_(Old/Young)| > 0.58.

Partial Least Squares-Discriminant Analysis (PLS-DA) revealed separated clustering of both native and ECM-enriched samples based on age, indicating that ovaries from reproductively young and old mice have distinct proteomic signatures (Figure 5A, 5B). Proteomic analysis identified 4,084 proteins in native mouse ovaries and 3,160 proteins in ECM-enriched mouse ovaries, of which 2,523 were present in both native and ECM-enriched samples (Supplementary Tables 1 and 2; Supplementary Figure 2). ECM-enriched ovaries had a different proteome from native counterparts, which in fact, included 637 proteins not detected in native samples (Supplementary Table 2; Supplementary Figure 2). The difference in the number of proteins identified between native and ECM-enriched ovaries was as anticipated, given the removal of cellular proteins in the ECM-enriched samples. All proteins in native and ECM-enriched samples were identified if ≥2 unique peptides per protein were detected in both reproductively young and reproductively old ovary samples. These proteins were subsequently quantified across all conditions using our data-independent acquisition approach and generating relative quantification results. We did not quantify changes between native and ECM-enriched samples as this was not the focus of our study.

Overall, 180 proteins were significantly altered (*q*-value < 0.01 and absolute average log_2_ ratio > 0.58) with advanced reproductive age in native ovaries (Supplementary Table 3), and 279 proteins were significantly altered with age in ECM-enriched ovaries (Supplementary Table 4), of which 76 proteins were common between groups (Supplementary Figure 2). The most highly upregulated proteins with advanced reproductive age included immunoglobulins, ECM proteins such as tenascin-C, tenascin-X, and TGFβ-induced protein, proteins that regulate metal homeostasis such as ferritin and ceruloplasmin, as well as tumor suppressors such as SDH enzyme and glycoprotein NMB (Figure 5C, 5D). Significantly downregulated proteins with age included oocyte-expressed protein homolog (OOEP) as well as NOD-, LRR-, and pyrin domain-containing proteins 5 and 14 and KH domain-containing protein 3 (Figure 5C, 5D). These proteins are expressed in the oocyte or are required for early embryonic development, and thus they are anticipated to decrease given the age-dependent loss of germ cells. The identification of oocyte-specific proteins, including OOEP, in ECM-enriched ovaries was surprising given the several wash steps with large volumes and agitation that follow detergent treatment in our decellularization protocol, which we would expect to remove most proteins from lysed cells. However, although the majority of H&E-stained stained tissue sections from ECM-enriched ovaries lacked visible nuclei, there were still some cells remaining, which may explain the detection of these cellular proteins. These conditions for decellularization were selected intentionally to enrich for the ECM, while still preserving the integrity of the matrix, which resulted in the tradeoff of some residual cells.

### Core matrisome and matrisome-associated proteins are significantly altered with age

To annotate core matrisome (collagens, proteoglycans, and ECM glycoproteins) and matrisome-associated proteins (ECM regulators, ECM-affiliated proteins, and secreted factors) in the mouse ovary, we utilized Matrisome DB, a database that compiles *in silico* and experimental data on matrisome proteins [39, 40]. We identified 81 core matrisome and 93 matrisome-associated proteins in native ovaries, as well as 74 core matrisome and 61 matrisome-associated proteins in ECM-enriched ovaries (Supplementary Table 5). Surprisingly, only seven matrisome proteins were specific to ECM-enriched samples, whereas 45 matrisome proteins were specific to native samples (Supplementary Table 5). Five secreted factors (anti-mullerian hormone, S100 calcium-binding protein A1, hepatocyte growth factor activator, secreted frizzled-related protein 1, and follistatin) were among the proteins specific to native ovaries (Supplementary Table 5). Growth factors are often sequestered in the ECM and the detection of some secreted factors in native ovaries that were not identified in ECM-enriched samples was anticipated given that detergent treatment and subsequent wash steps may remove some of the loosely bound proteins. However, eleven growth factors were identified in both native and ECM-enriched samples, further supporting that our conditions for ECM-enrichment did not perturb overall matrix integrity (Supplementary Table 5).

**Figure 6:**
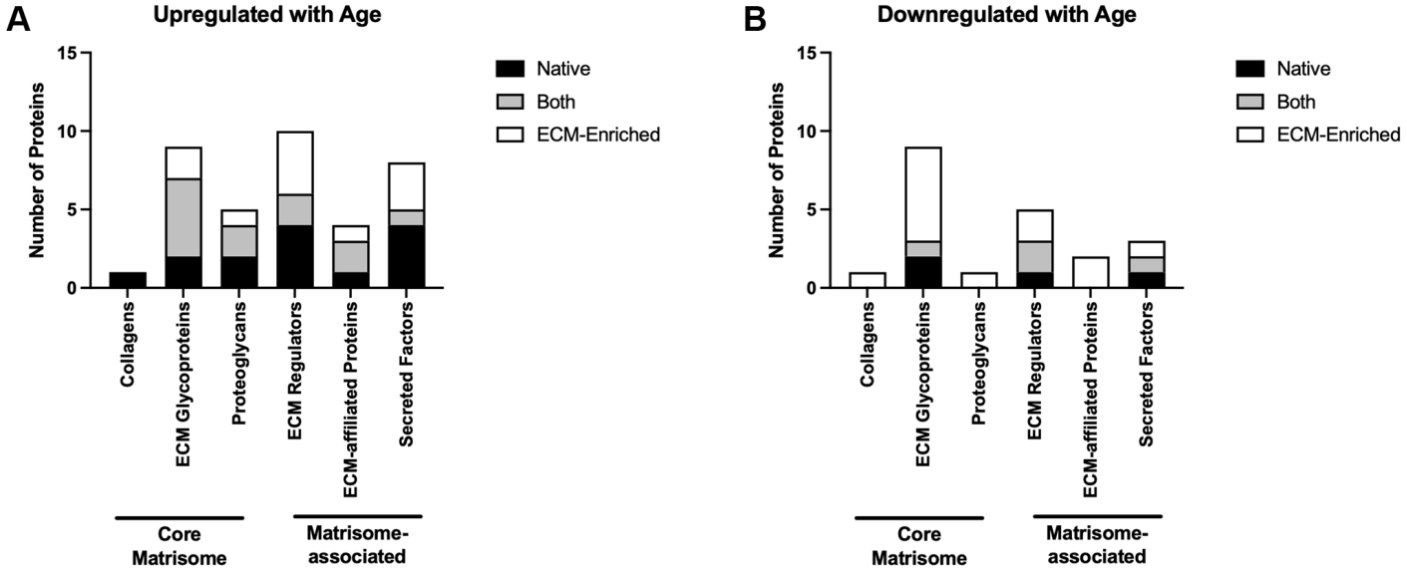
**Core matrisome and matrisome-associated proteins are significantly altered in the ovary with advanced reproductive age.** Quantification of core matrisome components (collagens, ECM glycoproteins, and proteoglycans) and matrisome-associated proteins (ECM regulators, ECM-affiliated proteins, and secreted factors) that are upregulated (**A**) and downregulated (**B**) in the ovary with advanced reproductive age.

Of the proteins that were upregulated with advanced reproductive age in either native or ECM-enriched samples or in both groups, 15 were core matrisome proteins and 22 were matrisome-associated proteins (Figure 6A: Supplementary Table 6). Eleven of the proteins downregulated in the ovary with advanced reproductive age were part of the core matrisome and ten were matrisome-associated proteins (Figure 6B; Supplementary Table 5). Overall, 14 of the core matrisome and matrisome-associated proteins upregulated with age were specific to the native ovary, 11 were specific to the ECM-enriched ovary, and 12 were present in both (Supplementary Table 6). Most of the core matrisome and matrisome-associated proteins downregulated with age were specific to the ECM-enriched ovary (13 proteins), with only four downregulated proteins specific to the native ovary and four proteins present in both samples (Supplementary Table 6). Of the 13 proteins downregulated with age specific to the ECM-enriched ovary, six of them were ECM glycoproteins including thrombospondin-1, fibrillin-2, and three zona pellucida proteins, which are specialized glycoproteins that surround the oocyte (Supplementary Table 6). Ovarian collagen, collagen VI α6, was only detected in the native ovary and was upregulated with age, whereas collagen XI α1 was distinctly detected in the ECM-enriched ovary and was downregulated with age (Supplementary Table 6). Overall, these data demonstrate that ECM proteins are some of the most robustly dysregulated in the ovary with advanced reproductive age.

To determine if the proteins upregulated with age in the mouse ovary may be implicated in age-associated ovarian fibrosis, we cross-referenced our dataset with FibroAtlas, a database of genes associated with fibrosis in several organ systems [41]. In fact, 17 of the matrisome proteins upregulated with age in the native ovary and 13 of the matrisome proteins upregulated with age in the ECM-enriched ovary have been previously associated with fibrosis in other organs (Table 1).

**Table 1:** Matrisome proteins upregulated with age previously associated with fibrosis.

**Protein**	**Protein description**	**UniProt accession**	**Average Log2 ratio**	***Q*-value**	**Experimental group**
DPT	Dermatopontin	Q9QZZ6	1.45	4.38E-07	Native
TGFBI	Transforming growth factor-beta-induced protein	P82198	0.87	8.20E-27	Native
CTSS	Cathepsin S	F6WR04; O70370	0.88	1.39E-04	Native
CTSC	Cathepsin C	P97821	0.75	4.04E-10	Native
CTSB	Cathepsin B	P10605	0.60	2.22E-14	Native
BGN	Biglycan	P28653	0.66	5.79E-07	Native
SFRP1	Secreted frizzled-related protein 1	Q8C4U3	0.82	5.22E-03	Native
FGF2	Fibroblast growth factor 2	P15655; Q925A1; Q925A2	0.78	6.10E-04	Native
SFRP4	Secreted frizzled-related sequence protein 4	Q9Z1N6	0.72	1.72E-04	Native
F9	Coagulation factor IX	P16294	1.51	1.10E-04	ECM-Enriched
HRG	Histidine-rich glycoprotein	A0A0R4J039; Q9ESB3	0.97	4.25E-07	ECM-Enriched
CTSL	Cathepsin L1	P06797	0.88	2.70E-04	ECM-Enriched
OGN	Mimecan	Q62000	0.70	9.44E-04	ECM-Enriched
TNC	Tenascin	Q80YX1	1.21	2.41E-82	Both
TNXB	Tenascin-X	O35452	0.73	1.33E-51	Both
MATN2	Matrilin-2	A0A0A0MQM7; O08746; O08746-2	0.88	4.40E-08	Both
POSTN	Periostin	Q62009; Q62009-2; Q62009-3; Q62009-4; Q62009-5	0.95	1.65E-06	Both
CTSD	Cathepsin D	P18242	0.58	2.65E-08	Both
MBL2	Mannose-binding protein C	P41317	1.32	6.90E-05	Both
DCN	Decorin	P28654	0.68	2.73E-07	Both
WNT4	Wnt-4	P22724	1.30	3.34E-03	Both

### Pathways associated with genomic stability and proteostasis are downregulated in the ovary with age

**Figure 7:**
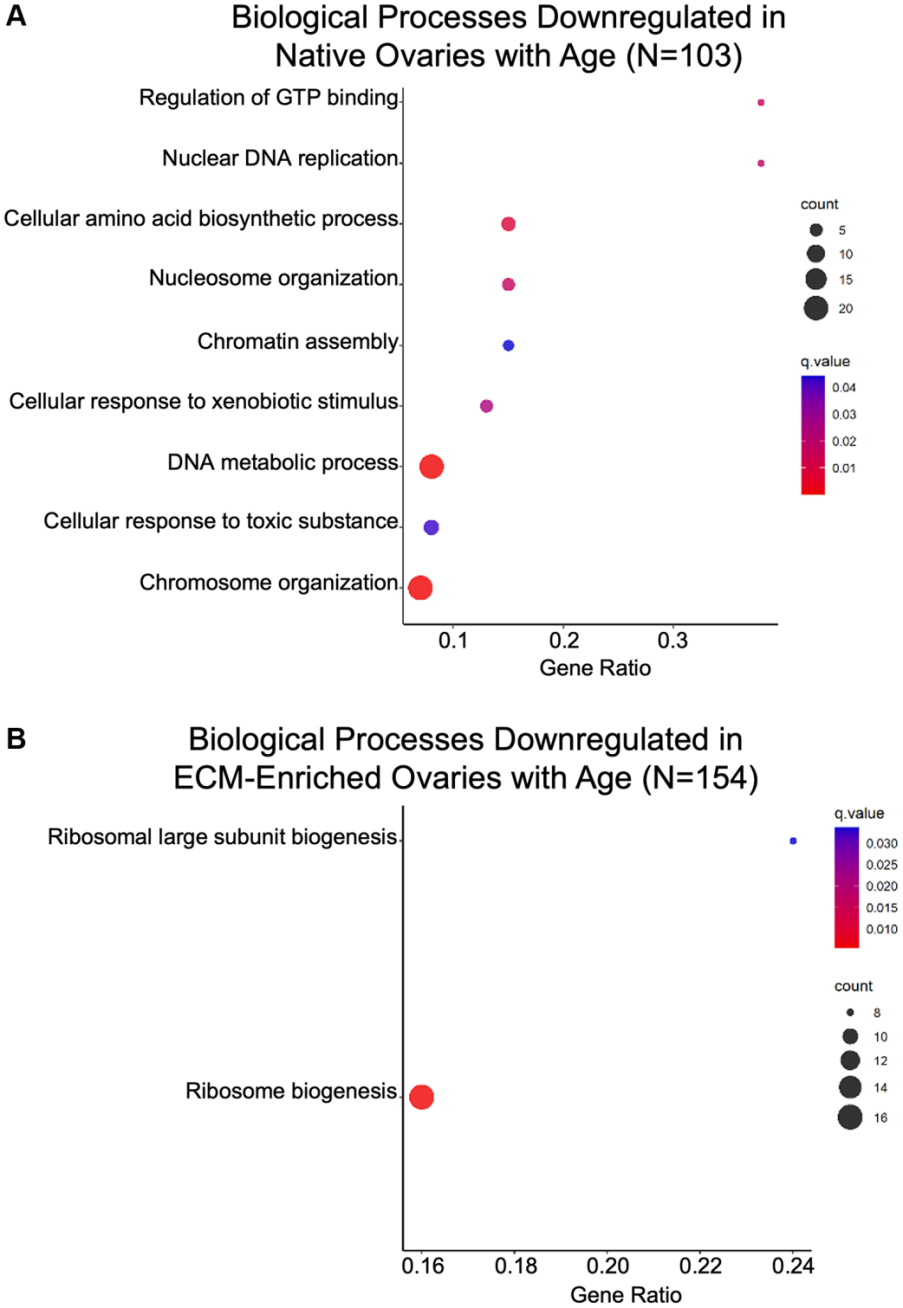
**Biological processes associated with genomic stability and proteostasis are downregulated in the ovary with age.** GO analysis of proteins significantly downregulated with age from (**A**) native and (**B**) ECM-enriched ovaries was performed using consensus pathway database (CPDB) at level 4, *q*-value < 0.05. The level of enrichment (corrected gene ratio) is depicted from 0–1, corresponding to 0–100% enrichment of the listed pathway.

Gene ontology analysis of proteins significantly altered with advanced reproductive age revealed that biological processes associated with genomic stability and epigenetic regulation, including “Nuclear DNA replication”, “Nucleosome organization”, “Chromatin assembly”, “DNA metabolic process”, and “Chromosome organization” were downregulated with age in native ovaries (Figure 7A; Table 2). Interestingly, several members of the mini-chromosome maintenance (MCM) family of DNA replication licensing factor proteins (MCM2, MCM3, MCM4, MCM6, and MCM7) drove these GO terms (Table 2). DNA replication, epigenetic modification, and DNA packaging-related molecular functions were also downregulated in both native and ECM-enriched ovaries with age (Supplementary Figure 3). GO terms associated with DNA helicase activity, DNA methyltransferase activity, and histone binding were downregulated in the ovary with age (Supplementary Figure 3). Moreover, biological processes associated with maintaining proteostasis, including “Cellular amino acid biosynthetic process” and “Ribosome biogenesis” were downregulated with age in both native and ECM-enriched ovaries (Figure 7A, 7B).

**Table 2:** Proteins driving biological processes associated with genomic stability and epigenetic regulation.

**Protein**	**Protein description**	**UniProt accession**	**Average Log2 ratio**	***Q*-value**
**Chromosome organization**				
PADI6	Protein-arginine deiminase type-6	Q8K3V4	−2.07	7.41E-46
KHDC3	Isoform 2 of KH domain-containing protein 3	Q9CWU5-2	−2.18	3.50E-12
NPM2	Nucleoplasmin-2	Q80W85	−2.12	4.61E-07
KPCD	Protein kinase C delta type	P28867; P28867-2	−0.68	6.91E-07
DNM3A	DNA (cytosine-5)-methyltransferase 3A	O88508	−0.84	1.56E-06
MKI67	Proliferation marker protein Ki-67	E9PVX6	−1.10	2.52E-03
**DNA metabolic process**				
MCM5	DNA helicase	Q52KC3	−1.01	4.12E-17
MCM3	DNA replication licensing factor MCM3	P25206	−0.70	8.26E-17
RFC3	Replication factor C subunit 3	Q8R323	−0.65	6.27E-03
**DNA metabolic process, Chromosome organization**				
UHRF1	E3 ubiquitin-protein ligase UHRF1	Q8VDF2; Q8VDF2-2	−1.42	2.75E-18
MCM6	DNA replication licensing factor MCM6	P97311; Q3ULG5	−0.81	1.69E-13
MCM7	DNA replication licensing factor MCM7	Q61881	−1.12	3.05E-11
DNMT1	DNA (cytosine-5)-methyltransferase 1	P13864	−0.63	6.94E-10
MCM4	DNA replication licensing factor MCM4	P49717	−1.16	5.80E-08
CDK1	Cyclin-dependent kinase 1	P11440	−1.14	6.28E-06
TOP2A	DNA topoisomerase 2-alpha	Q01320	−1.34	1.25E-04
SMC4	Structural maintenance of chromosomes protein	E9Q2X6; Q8CG47	−1.17	2.45E-03
EXOS	Exosome component 10	P56960; Q8K366	−0.58	3.24E-03
**Nuclear DNA replication, Chromosome organization**				
PCNA	Proliferating cell nuclear antigen	P17918	−0.71	4.49E-06
**Nuclear DNA replication, DNA metabolic process**				
WIZ	Widely-interspaced zinc finger containing protein	F6ZBR8	−0.60	8.07E-05
DNLI1	DNA ligase 1	P37913; Q3U4X8	−0.98	3.37E-03
**Nucleosome organization, Chromosome organization**				
NP1L5	Nucleosome assembly protein 1-like 5	Q9JJF0	−0.77	3.41E-06
HAT1	Histone acetyltransferase type B catalytic subunit	A2ATU9; Q8BY71	−0.93	2.57E-04
PTMA	Prothymosin alpha	P26350	−0.59	3.26E-03
**Nucleosome organization, DNA metabolic process, Chromosome organization**				
MCM2	DNA replication licensing factor MCM2	P97310	−1.05	3.13E-16
NASP	Nuclear autoantigenic sperm protein	B1AU75; Q99MD9	−0.62	3.70E-14

### Pathways associated with inflammation and ECM organization are upregulated in the ovary with age

**Figure 8:**
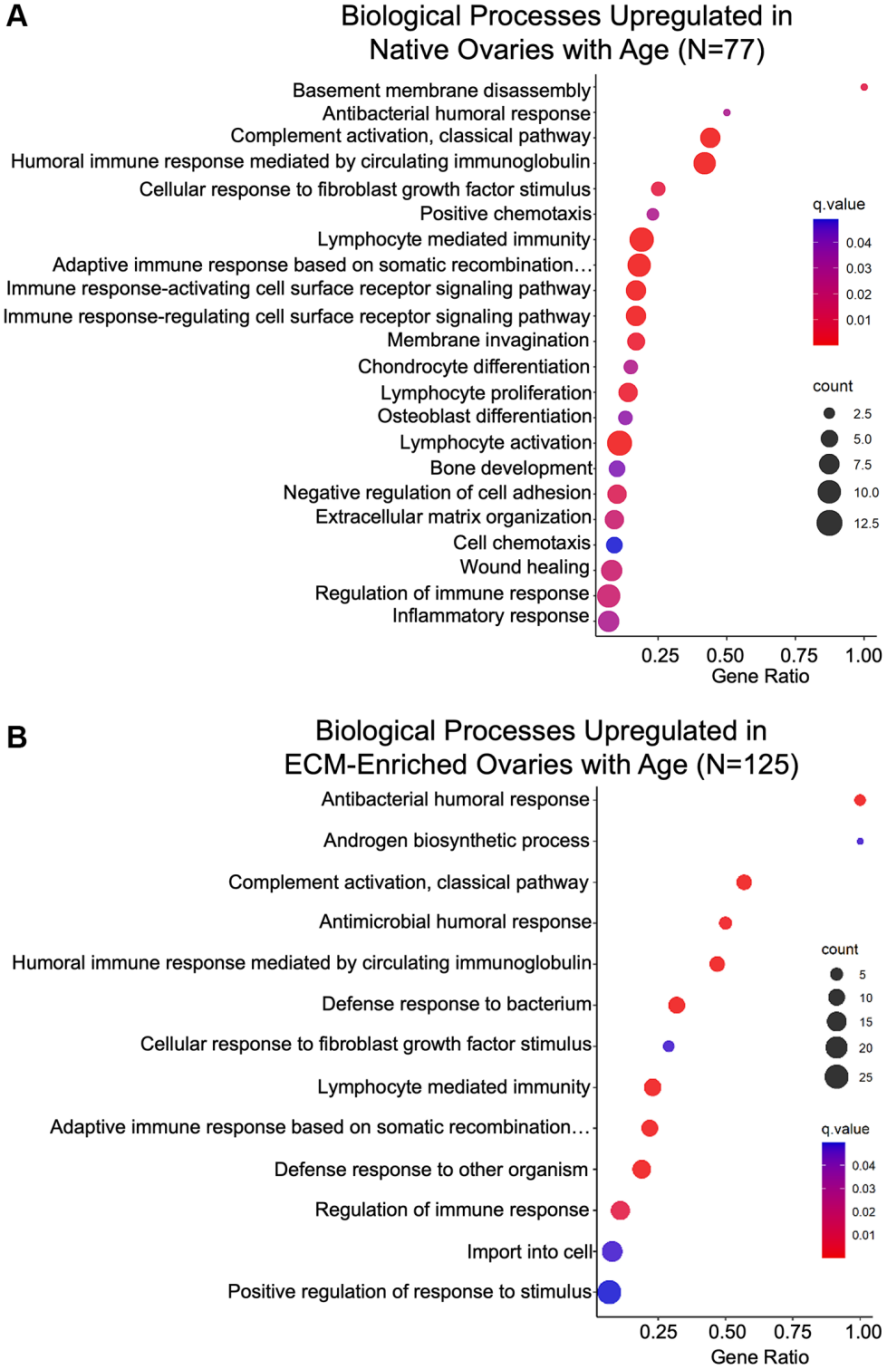
**Biological processes associated with inflammation and ECM organization are upregulated in the ovary with age.** GO analysis of proteins significantly upregulated with age from (**A**) native and (**B**) ECM-enriched ovaries was performed using consensus pathway database (CPDB) at level 4, *q*-value < 0.05. The level of enrichment (corrected gene ratio) is depicted from 0–1, corresponding to 0–100% enrichment of the listed pathway.

Biological processes associated with immune response were enriched in gene ontology analysis of proteins significantly altered with age in native and ECM-enriched ovaries (Figure 8A, 8B; Table 3). These upregulated immune-related pathways included “Antibacterial humoral response”, “Complement activation”, “Humoral response mediated by circulating immunoglobulins”, “Lymphocyte mediated immunity”, and “Regulation of immune response” (Figure 8A, 8B; Table 3). Age-associated upregulation of “Complement activation” is interesting given the complement system has been implicated in several age-related diseases, including Alzheimer’s disease and age-related macular degeneration [42]. In the ovary, however, the majority of research on the complement cascade has been done in the context of ovarian cancer [43]. Several immunoglobulins, complement component 4b (C4B), complement component receptor 1-like protein (CR1L), and the mitochondrial protein complement C1q binding protein (C1QBP) are among proteins that drive this GO term (Table 3).

**Table 3:** Proteins driving biological processes associated with immune response.

**Protein**	**Protein description**	**UniProt accession**	**Average Log2 ratio**	***Q*-value**	**Experimental group**
**Antibacterial humoral response; Complement activation, classical pathway; Humoral immune response mediated by circulating immunoglobulin; Lymphocyte mediated immunity; Adaptive immune response based on somatic recombination…; Regulation of immune response; Inflammatory response (native only)**					
IGHG1	Immunoglobulin heavy constant gamma 1	A0A075B5P4; A0A0A6YWR2	2.02	6.00E-04	Both
IGHG2C	Immunoglobulin heavy constant gamma 2C	A0A0A6YY53; F6TQW2	1.91	7.99E-04	Both
**Antibacterial humoral response**					
FGB	Fibrinogen beta chain	Q8K0E8	0.60	6.84E-17	ECM-Enriched
FGA	Fibrinogen alpha chain	E9PV24; E9PV24-2	0.59	1.48E-10	ECM-Enriched
**Complement activation, classical pathway; Humoral immune response mediated by circulating immunoglobulin; Lymphocyte mediated immunity; Adaptive immune response based on somatic recombination…; Regulation of immune response**					
IGHM	Immunoglobulin heavy constant mu	A0A075B5P6; A0A075B6A0; P01872; P01872-2	1.50	1.19E-18	Both
MBL2	Mannose-binding protein C	P41317	1.15	1.26E-06	Both
IGHG2B	Immunoglobulin heavy constant gamma 2B	A0A075B5P3	1.49	4.52E-11	Native
IGHV1-5	Immunoglobulin heavy variable V1-5	A0A075B5T5	2.46	2.50E-05	Native
IGHV1-15	Immunoglobulin heavy variable V1-15	A0A0A6YXA5	1.87	2.04E-03	Native
APCS	Serum amyloid P-component	P12246	0.59	2.96E-03	Native
CR1L	Complement component receptor 1-like protein	Q64735; Q64735-2	0.73	1.35E-05	ECM-Enriched
C1QBP	Complement component 1Q-binding protein	Q8R5L1	0.72	5.57E-04	ECM-Enriched
C4B	Complement component 4B	P01029	0.70	2.90E-03	ECM-Enriched
IGHV1-31	Immunoglobulin heavy variable V1-31	A0A075B5V1	1.39	7.61E-03	ECM-Enriched
**Humoral immune response mediated by circulating immunoglobulin; Lymphocyte mediated immunity; Adaptive immune response based on somatic recombination…; Regulation of immune response**					
PTPRC	Protein tyrosine phosphatase receptor type C	P06800; P06800-2; P06800-3; S4R1M0; S4R1S4; S4R2V1	1.42	1.47E-06	Native
PTPN6	Protein tyrosine phosphatase non-receptor type 6	P29351; P29351-2; P29351-3	0.62	2.86E-05	Native
**Lymphocyte mediated immunity; Adaptive immune response based on somatic recombination…**					
CTSC	Cathepsin C	P97821	0.75	4.04E-10	Native
**Lymphocyte mediated immunity**					
CORO1A	Coronin-1A	O89053	0.60	5.93E-09	Native
**Lymphocyte mediated immunity; Regulation of immune response**					
LAMP1	Lysosome-associated membrane glycoprotein 1	P11438	0.61	8.39E-06	ECM-Enriched
**Lymphocyte mediated immunity; Adaptive immune response based on somatic recombination…; Regulation of immune response**					
B2M	Beta-2-microglobulin	P01887	0.76	1.86E-03	ECM-Enriched
CD74	CD74 antigen	P04441; P04441-2	0.90	4.67E-03	ECM-Enriched
**Regulation of immune response**					
MNDAL	Myeloid cell nuclear differentiation antigen-like protein	D0QMC3	0.64	2.58E-03	Native
APOE	Apolipoprotein E	P08226	2.01	3.82E-14	ECM-Enriched
FLOT1	Flotillin-1	O08917	0.60	1.08E-11	ECM-Enriched
HRG	Histidine-rich glycoprotein	A0A0R4J039; Q9ESB3	0.97	4.25E-07	ECM-Enriched
**Inflammatory response (native only)**					
NT5E	5′-nucleotidase	Q61503	1.46	7.17E-08	Both
FABP4	Fatty acid-binding protein 4	P04117	3.09	8.75E-06	Native
HP	Haptoglobin	Q61646	2.62	1.70E-05	Native
CLU	Clusterin	Q06890	0.61	2.23E-05	Both
CTSS	Cathepsin S	F6WR04; O70370	0.88	1.39E-04	Native
SAA4	Serum amyloid A-4 protein	P31532	1.03	2.55E-04	Native
LIPA	Lysosomal acid lipase A	Q9Z0M5	1.20	5.09E-04	Native

Increased inflammation in the aging ovary may be in part due to increased oxidative stress and consistent with this, molecular functions associated with oxidoreductase activity were upregulated with age (Supplementary Figure 4) [44–46]. Chronic inflammation in the aging ovary has been associated with the presence of a unique population of multinucleated macrophage giant cells (MNGCs) [13, 47]. Although these ovarian MNGCs have not been well characterized, osteoclasts are one of three main subtypes of MNGCs [48]. Thus, upregulation of “Chondrocyte differentiation”, “Osteoblast differentiation”, and “Bone development” in the native ovary with age may be indicative of MNGC signaling and proteins that underlie these GO terms including transmembrane glycoprotein NMB (GPNMB) and transforming growth factor-β-induced protein (TGFBI) may be putative markers for ovarian MNGCs (Figure 8A; Table 4). Macrophage fusion to form MNGCs allows for enhanced phagocytosis and the “Membrane invagination” biological process was upregulated in the native ovary with age, consistent with this potential increase in phagocytic activity (Figure 8A; Table 4) [49].

**Table 4:** Proteins driving biological processes putatively associated with multi-nucleated macrophage giant cells.

**Protein**	**Protein description**	**UniProt accession**	**Average Log2 ratio**	***Q*-value**
**Chondrocyte differentiation**				
TGFBI	Transforming growth factor-beta-induced	P82198	0.87	8.20E-27
EFEMP1	EGF-containing fibulin-like extracellular matrix protein 1	Q8BPB5	1.18	6.05E-13
**Chondrocyte differentiation; Bone development**				
MATN2	Matrilin-2	A0A0A0MQM7; O08746; O08746-2	0.88	4.40E-08
MBL2	Mannose-binding protein C	P41317	1.15	1.26E-06
**Osteoblast differentiation**				
GPNMB	Transmembrane glycoprotein NMB	Q8BVA0; Q99P91	1.96	1.69E-06
FGF2	Fibroblast growth factor 2	P15655; Q925A1; Q925A2	0.78	6.10E-04
WNT4	Wnt-4	P22724	1.43	1.68E-03
SFRP1	Secreted frizzled-related protein 1	Q8C4U3	0.82	5.22E-03
**Bone development**				
PTPRC	Protein tyrosine phosphatase receptor type C	P06800; P06800-2; P06800-3; S4R1M0; S4R1S4; S4R2V1	1.42	1.47E-06
PTPN6	Protein tyrosine phosphatase non-receptor type 6	P29351; P29351-2; P29351-3	0.62	2.86E-05
SFRP4	Secreted frizzled-related sequence protein 4	Q9Z1N6	0.72	1.72E-04
**Membrane invagination**				
IGHM	Immunoglobulin heavy constant mu	A0A075B5P6; A0A075B6A0; P01872; P01872-2	1.50	1.19E-18
IGHG1	Immunoglobulin heavy constant gamma 1	P01868; P01869	1.78	8.52E-16
IGHG2B	Immunoglobulin heavy constant gamma 2B	A0A075B5P3	1.49	4.52E-11
IGHV1-5	Immunoglobulin heavy variable V1-5	A0A075B5T5	2.46	2.50E-05
IGHV1-15	Immunoglobulin heavy variable V1-15	A0A0A6YXA5	1.87	2.04E-03
IGHG2C	Immunoglobulin heavy constant gamma 2C	A0A0A6YY53; F6TQW2	2.98	2.93E-03

Inflammation often precedes fibrosis, and biological processes relevant to ECM remodeling and fibrosis were also upregulated with age, including “Extracellular matrix organization” and “Wound healing” in native ovaries, as well as “Cellular response to fibroblast growth factor stimulus” in both native and ECM-enriched ovaries (Figure 8A; Table 5). Although age-associated ovarian fibrosis has been well characterized, the mechanisms that underlie this fibrosis are not well understood and further research is required to determine how proteins including tenascin-C (TNC), cathepsin family members, and transforming growth factor-β-induced protein (TGFBI), which drive fibrosis-related GO terms may contribute to this robust ovarian aging phenotype (Table 5). Binding of core matrisome components and matrisome-associated proteins, including syndecans, proteoglycans, collagens, and glycosaminoglycans, were among molecular functions upregulated with age in both native and ECM-enriched ovaries (Supplementary Figure 4). Age-associated fibrosis in the ovary contributes to increased tissue stiffness, and consistent with this, the “Extracellular matrix constituent conferring elasticity” pathway was downregulated in native ovaries from reproductively old mice (Supplementary Figure 3) [19, 21].

**Table 5:** Proteins driving biological processes associated with ECM remodeling.

**Protein**	**Protein description**	**UniProt accession**	**Average Log2 ratio**	***Q*-value**	**Experimental group**
**Cellular response to fibroblast growth factor stimulus; Extracellular matrix organization (native only); Wound healing (native only)**					
POSTN	Periostin	Q62009; Q62009-2; Q62009-3; Q62009-4; Q62009-5	0.95	1.65E-06	Both
**Cellular response to fibroblast growth factor stimulus; Wound healing (native only)**					
WNT4	Wnt-4	P22724	1.43	1.68E-03	Both
FGF2	Fibroblast growth factor 2	P15655; Q925A1; Q925A2	0.78	6.10E-04	Native
**Cellular response to fibroblast growth factor stimulus**					
SFRP1	Secreted frizzled-related protein 1	Q8C4U3	0.82	5.22E-03	Native
NGFR	Nerve growth factor receptor	Q8CFT3; Q9Z0W1	1.26	3.54E-03	ECM-Enriched
GPC1	Glypican-1	Q9QZF2	0.85	5.23E-03	ECM-Enriched
**Extracellular matrix organization (native only)**					
TGFBI	Transforming growth factor-beta-induced	P82198	0.87	8.20E-27	Both
DPT	Dermatopontin	Q9QZZ6	1.45	4.38E-07	Both
TNXB	Tenascin-X	O35452	0.73	1.33E-51	Native
LUM	Lumican	P51885	0.68	7.91E-13	Native
CTSS	Cathepsin S	F6WR04; O70370	0.88	1.39E-04	Native
CMA1	Chymase	A4QPC5; P21844	1.88	6.73E-03	Native
**Wound healing (native only)**					
TNC	Tenascin	Q80YX1	1.21	2.41E-82	Both
DCN	Decorin	P28654	0.97	7.04E-09	Both
CASK	Calcium/calmodulin-dependent serine protein kinase	A0A067XG53; O70589; O70589-3; O70589-4; O70589-5	0.66	2.48E-07	Native
SERPIND1	Heparin cofactor 2	P49182	0.86	1.04E-06	Native
PTPN6	Protein tyrosine phosphatase non-receptor type 6	P29351; P29351-2; P29351-3	0.62	2.86E-05	Native
EPPK1	Epiplakin	Q8R0W0	0.61	1.58E-03	Native

## DISCUSSION

**Figure 9:**
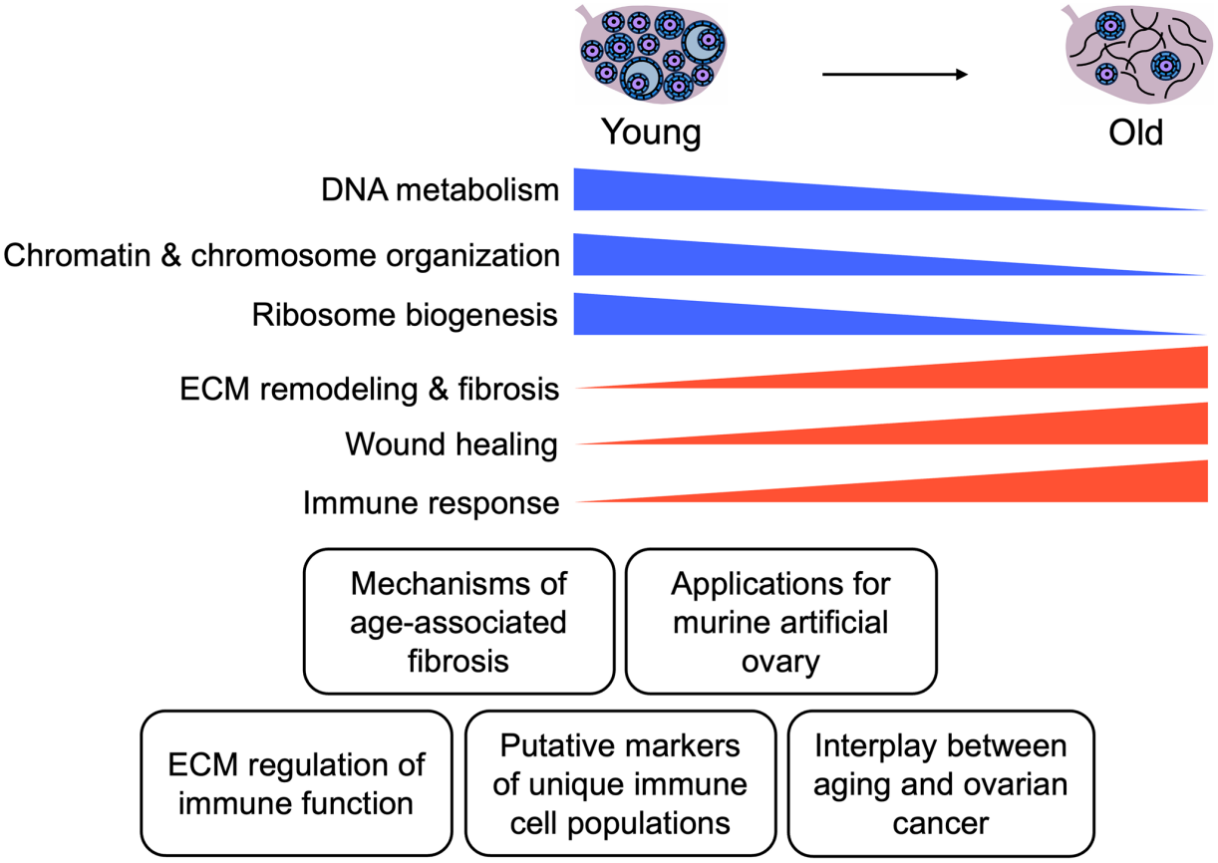
**Summary of key findings.** Schematic of key pathways significantly altered in the mouse ovary with age and potential applications of these data for future studies.

In this study, we established an effective ECM enrichment strategy for the mouse ovary across reproductive age that removes most cellular content, as demonstrated by loss of nuclear staining in ovarian tissue sections and lack of detectable DNA bands on agarose gels, while still preserving the collagen matrix. This approach enabled the interrogation of the proteome of ECM-enriched ovaries in parallel with native ovaries. The native and ECM-enriched samples clustered based on animal age, indicating distinct age-dependent ovarian proteomic signatures. The cell composition in reproductively young and reproductively old ovaries is inherently different and likely underlies the age-dependent proteomic signatures we observed. Several of the proteins that were significantly altered with advanced age were core matrisome components or matrisome-associated proteins. Interestingly, many of the matrisome proteins upregulated in the ovary with age have been implicated in fibrosis in other organs (Figure 9). Pathways associated with genomic stability and epigenetic regulation were downregulated with age, whereas pathways associated with immune response and ECM remodeling were upregulated (Figure 9). Taken together, our results inform how the aging ovarian microenvironment, including age-associated changes in cell composition, impact the ovarian proteome and matrisome.

To our knowledge, this is the first study to utilize unbiased proteomic approaches to investigate the effect of reproductive aging on the murine ovarian proteome and matrisome. However, several other groups have utilized proteomic methods to characterize the composition of the ovary, as well as ovarian structures and cell types, including antral follicles, corpora lutea, and granulosa cells from various species [26, 34, 50–58]. Studies that profiled fetal mouse ovaries, mouse ovaries in the context of the pubertal transition, or mouse ovarian follicles identified proteins implicated in steroidogenesis, the cell cycle, the complement cascade, ECM-receptor interaction, and metabolic regulation including glycolysis and the electron transport chain [56–58]. Similar to these previous studies, we found enrichment of proteins involved in ECM binding, complement activation, DNA replication and metabolism, as well as electron transport chain activity. This conservation of pathways across datasets indicates that these processes are essential for ovarian function throughout the reproductive lifespan. However, pathways involved in steroidogenesis were not as highly enriched in our data, as many steroidogenic enzymes did not meet our stringent filtering criteria. Moreover, previous reports have characterized the ovarian matrisome in human and porcine models [34, 50, 59]. These studies identified more than 80 ovarian matrisome proteins divided among 6 categories (collagens, ECM glycoproteins, ECM regulators, ECM-affiliated proteins, proteoglycans, and secreted factors) [26, 34, 50, 59]. Here, we add to current knowledge in the field by characterizing the murine ovarian matrisome, and we identified 82 matrisome proteins previously not identified in proteomic studies of human or porcine ovaries (Supplementary Table 5) [26, 34, 50, 59].

The mouse is a tightly controlled model system and is ideal for studying age-dependent changes to the ovarian proteome because analysis can be performed on whole ovaries, providing an understanding of how reproductive aging affects all ovarian sub-compartments, such as follicles, corpora lutea, and stroma. Nevertheless, there has been a previous proteomic study of human ovarian cortical biopsies which identified 26 matrisome proteins with significantly altered expression across three age cohorts (pre-puberty, reproductive-age, and menopause) [26]. Interestingly, differentially expressed ECM proteins in the human ovarian cortex post-menopause contribute to upregulation of pathways associated with ECM polymerization, ROS generation, cell migration, cell damage and tissue repair [26]. Although these pathways were similarly upregulated with age in both the native and ECM-enriched mouse ovary, most of the specific proteins driving these processes in the human were not significantly altered in the mouse (Figure 8).

In addition to profiling native ovaries from reproductively young and old mice, we enriched for the ECM prior to proteomic analysis to examine the effect of reproductive aging specifically on the ovarian matrix. ECM-enrichment allowed for the identification of 7 additional matrisome proteins not present in native samples, as well as 24 additional matrisome proteins that were significantly altered with age (Figure 6; Supplementary Tables 5, 6). Interestingly, 40 of the 279 proteins that were significantly altered with age in ECM-enriched ovaries were core matrisome components or matrisome-associated proteins, and in fact proteins that were significantly upregulated with age in both native and ECM-enriched ovaries were related to immune response and ECM remodeling pathways (Figure 6; Supplementary Table 6; Figure 8). Although these similarities between native and ECM-enriched ovaries may be in part explained by incomplete decellularization, this is unlikely to be the case given that the majority of the ECM-enriched tissue lacked nuclear and cellular contents. Instead, these data suggest that ECM proteins are among the most robustly impacted by ovarian aging and that there is likely important interplay between the ECM and immune system in the ovary that contributes to the age-dependent decline in function.

Macrophages are the most abundant immune cells in the ovary [17]. With advanced reproductive age, tissue-resident macrophages are depleted and are replaced by monocyte-derived macrophages, which are recruited to the ovary due to increased chemokine expression [17, 18]. In other organ systems, biochemical and biomechanical properties of the ECM influence the migration and infiltration of immune cells [60]. Thus, it is possible that the altered ECM composition and structure of the aging ovary facilitate increased macrophage infiltration. In fact, increased matrix crosslinking promotes macrophage migration in *in vitro* studies [61]. Moreover, increased monocyte-derived macrophage infiltration may contribute to ovarian fibrosis. Monocytes that migrate to fibrotic lungs transform into macrophages and remain in the lungs with high profibrotic gene expression [62]. In addition to shifted macrophage ontogeny, macrophages in ovaries from reproductively old mice fuse to form a unique population of multinucleated macrophage giant cells (MNGCs) [13]. These MNGCs are hallmarks of tissues with chronic inflammation, and in general there are three subtypes of MNGCs, one of which is osteoclasts [48]. Osteoclasts are bone resorbing cells, which participate in bone remodeling under physiologic conditions [48, 49]. However, under pathologic conditions including cancer metastasis to bone, rheumatoid arthritis, and osteoporosis, osteoclast activity contributes to bone destruction and loss [48, 49]. A driving factor of how aggressively osteoclasts remove bone volume is nuclei number [49]. Therefore, enrichment of biological processes including “Osteoblast differentiation” and “Bone development” following GO analysis of proteins upregulated with advanced age may be related to macrophage fusion into osteoclast-like MNGCs. In fact, transmembrane glycoprotein NMB (GPNMB) is upregulated in the ovary with age and is one of the proteins that drives the “Osteoblast differentiation” GO term (Table 4). GPNMB positive macrophages show enhanced phagocytic activity, which is characteristic of MNGCs [63]. Moreover, GPNMB promotes macrophage M2 polarization, which may contribute to known age-associated macrophage polarization in the ovary [17, 64]. Similarly, increased transforming growth factor-β-induced protein (TGFBI) expression underlies the “Chondrocyte differentiation” GO term that is upregulated in the aging mouse ovary (Table 4). Monocyte-derived macrophages produce TGFBI following ingestion of apoptotic cells, which ultimately contributes to increased collagen production by co-cultured fibroblasts in *in vitro* studies [65]. These proteins along with the others that drive the bone related GO terms upregulated with age, may be putative markers for ovarian MNGCs. Additional studies are necessary to further characterize these age-associated ovarian MNGCs, as well as to determine the functional effects of these MNGCs on ovarian aging.

In addition to macrophage-related pathways, “complement activation, classical pathway” is another immune-related GO term that was upregulated with age in both native and ECM-enriched ovaries in our study (Figure 8). Complement effectors can induce ROS production, as well as cytokine secretion, ultimately resulting in an inflammatory response [66, 67]. Future research is necessary to determine how complement activation contributes to inflammaging in the ovary. Complement C1q binding protein (C1QBP) was upregulated in the ECM-enriched ovary with age. C1QBP is a mitochondrial protein that regulates mitochondrial oxidative phosphorylation [68]. Given that mitochondrial function is compromised in the aging ovary, increased C1QBP protein expression may be a compensatory mechanism. On the other hand, the complement system can have negative impacts on mitochondria as exposure to complement C5a *in vitro* results in ROS production and impaired mitochondrial respiration of renal tubular epithelial cells [69]. Thus, it is also possible that upregulated complement activation in the aging ovary contributes to impaired mitochondrial function in the ovary with age. Furthermore, addition of C1Q promotes fibroblast proliferation and production of collagen through the Wnt signaling pathway [70]. As expression of Wnt signaling proteins (Wnt-4, secreted frizzled-related protein 1, and secreted frizzled-related protein 4) are increased in the ovary with age in our dataset and increased age-associated ovarian collagen production has been previously demonstrated, a role of the complement system in regulating these phenotypes warrants future investigation [13, 14, 16, 20].

Age-associated ovarian fibrosis has recently been proposed as a risk factor for ovarian cancer [16, 59, 71, 72]. Several of the ECM proteins we identified to be upregulated in the ovary have been previously associated with fibrosis in other organs and have been associated with a poor prognosis in ovarian cancer (Table 1). However, the mechanisms by which these proteins contribute to ovarian fibrosis or ovarian cancer pathogenesis are not well understood. For example, tenascin-C (TNC) stimulates collagen gene expression and activation of skin fibroblasts *in vitro* through toll-like receptor 4 signaling [73]. Knockout of TNC in a hepatitis mouse model attenuated fibrotic and inflammatory responses in the liver [73, 74]. TNC expression is increased in malignant ovarian tumors compared to benign tissue and high serum TNC levels in ovarian cancer patients has been correlated with poor overall survival [75, 76]. Moreover, ovarian cancer cells had increased adhesion and migration on TNC coated plates compared to controls [75]. Similarly, cathepsins B, C, D, and S were upregulated with age in our dataset and have all been implicated in liver or pulmonary fibrosis (Table 1) [77, 78]. Expression of cathepsins B and D is elevated in ovarian cancer and expression of a different cathepsin family member, cathepsin L, increases the invasion and metastatic capacity of ovarian cancer cells [79]. Another protein upregulated in native mouse ovaries with age, TGFBI, regulates pulmonary fibrosis and is secreted by ovarian cancer tumor-associated macrophages, promoting cancer cell migration [80, 81]. Consistent with some of ECM proteins that were upregulated in the aging ovary having a role in enhancing cancer cell migration, biological processes associated with cell migration including “Positive chemotaxis”, “Negative regulation of cell adhesion”, and “Cell chemotaxis” were enriched in native ovaries from reproductively old mice (Figure 8). Cancer cell migration is increased on fibrotic and stiff tissues [82–84]. Further studies are needed to examine how these proteins may contribute to age-related ovarian fibrosis and whether they impact ovarian cancer pathogenesis through their fibrotic functions or other mechanisms.

Overall, our study provides novel insight into how reproductive aging impacts the murine ovarian proteome and ECM. Our data highlight perturbation to pathways involved in ECM function, genomic stability, and inflammation, which have been previously demonstrated to be impaired with advanced age (Figure 9). However, we uncover specific proteins that drive these pathways, which are important targets for future mechanistic studies (Figure 9). Understanding how the ovarian microenvironment changes with age is an important first step in the development of therapeutic interventions, including the artificial ovary, to extend fertility and endocrine function.

## MATERIALS AND METHODS

### Animals

All experiments were performed using female CD-1 mice purchased from Envigo (Indianapolis, IN, USA) except for a small subset of optimization experiments which were performed with female CB6F1 purchased from Envigo (Supplementary Figure 1). Reproductively young mice were used for experiments at 6–12 weeks and reproductively old mice were used at 10–12 months of age. Reproductively young mice were virgin animals, whereas our reproductively old mice were retired breeders due to the resources required to age animals for this amount of time. At 10–12 months of age mice display reproductive aging phenotypes, including subfertility, fibrotic foci in the ovarian stroma, reduced follicle quantity, and decreased chromosome cohesion [13, 85–87]. Mice were housed in a controlled barrier facility at Northwestern University’s Center for Comparative Medicine (Chicago, IL, USA) under constant temperature, humidity, and light (14 h light/10 h dark). Upon arrival to Northwestern University, animals were provided water and Teklad Global irradiated 2016 chow (Envigo, Madison, WI, USA) containing minimal phytoestrogens, ad libitum. All mice were allowed to acclimate for a minimum of one week upon arrival in our vivarium prior to experimental use. All experiments were performed under protocols approved by the Institutional Animal Care and Use Committee (Northwestern University) and in accordance with the National Institutes of Health Guidelines for the Care and Use of Laboratory Animals.

### ECM enrichment

To enrich for the ECM, ovaries were washed in Dulbecco’s Phosphate-Buffered Saline (DPBS, Gibco, Grand Island, NY, USA) and immersed in DPBS containing 0.1% (w/v) sodium dodecyl sulfate (SDS, Sigma-Aldrich, St. Louis, MO, USA) for 12.5 hours, or specific times as described in the figure legends, at room temperature on a nutating rocker. Ovaries were then washed in 15 mL RO water (4 × 30 minutes) and 1 mL DPBS (1 × 1 hour) and incubated in DPBS containing 40 units/mL Deoxyribonuclease I (Sigma-Aldrich, St. Louis, MO, USA) for 30 min at 37°C. Ovaries were subsequently washed in 15 mL DPBS (2 × 30 minutes) and flash-frozen on dry ice or fixed in Modified Davidson’s (Electron Microscopy Sciences, Hatfield, PA, USA) overnight at 4°C. ECM enrichment in Supplementary Figure 1 was performed in DPBS containing 0.5 or 0.1% (w/v) SDS or 1% Triton X-100 (TX-100, Alfa Aesar, Haverhill, MA, USA) following the same procedure. Native ovaries were washed in DPBS and flash-frozen on dry ice or fixed in Modified Davidson’s overnight at 4°C immediately after harvesting.

To validate ECM-enrichment by measuring residual double stranded DNA, flash-frozen ovaries were lyophilized (Freezone 6, Labconco, Kansas City, MO, USA) at the Northwestern University Analytical bioNanoTechnology Core Facility (ANTEC). DNA was extracted from lyophilized ovaries using a Quick-DNA MidiPrep Plus Kit (Zymo Research, Irvine, CA, USA) following the manufacturer’s instructions. Following extraction, DNA was run on a 1% (w/v) agarose gel with a 1 kb DNA ladder (New England Biolabs, Ipswich, MA, USA). Agarose gels were imaged using a Bio-Rad ChemiDoc Imaging System (Bio-Rad, Hercules, CA, USA).

### Tissue processing and histochemical staining

Following fixation, native and ECM-enriched ovaries were transferred to 70% ethanol and stored at 4°C until processing. Samples were dehydrated using an automated tissue processor (Leica Biosystems, Buffalo Grove, IL, USA), embedded in paraffin, and sectioned (5 μm thickness) using a Reichert-Jung Biocut 2035 microtome (Leica Biosystems, Buffalo Grove, IL, USA).

Hematoxylin and eosin (H&E) staining was performed using a Leica Autostainer XL (Leica Biosystems, Buffalo Grove, IL, USA). Tissue sections were cleared with Xylene (Mercedes Scientific, Lakewood Ranch, FL, USA) in 3, 5-minute incubations and mounted with Cytoseal XYL (ThermoFisher Scientific, Waltham, MA, USA). Hematoxylin staining was performed following a standard protocol. Tissue sections were cleared with Citrosolv (Fisher Scientific Pittsburgh, PA, USA) in 3, 5-minute incubations and mounted with Cytoseal XYL.

Picrosirius Red (PSR) staining was performed as previously published [13, 19]. Briefly, tissue sections were deparaffinized in Citrosolv, rehydrated in graded ethanol baths (100, 70, and 30%) and washed in RO water. Slides were immersed in PSR staining solution for 40 minutes, then incubated in acidified water (0.05 M hydrochloric acid) for 90 seconds. Tissue sections were dehydrated in 100% ethanol, cleared in Citrosolv, and mounted with Cytoseal XYL.

For Hoechst staining, tissue sections were deparaffinized in Citrosolv (2, 3-minute incubations), rehydrated in graded ethanol baths (100, and 95%) and washed in RO water and PBS. Slides were incubated with Hoechst (NucBlue, Invitrogen, Carlsbad, CA, USA; 2 drops per mL PBS) for 30 minutes in a humidified chamber. Slides were washed in PBS and mounted in Vectashield antifade mounting medium (Vector Laboratories, Burlingame, CA, USA).

### Imaging and image analysis

Brightfield images were taken with an EVOS FL Auto Cell Imaging System (ThermoFisher Scientific, Waltham, MA, USA) using a 10×, 20×, or 40× objective. To view entire ovarian tissue sections, scans comprised of a series of individual images were taken across the tissue and then automatically stitched together using the EVOS software. Epifluorescence images were taken with an EVOS FL Auto Cell Imaging System equipped with a DAPI LED light cube (Excitation 357/44 nm, Emission 447/60 nm) using a 10× or 40× objective. Imaging settings, including light, gain, and exposure times, were kept consistent between samples.

Image processing and analysis was performed using FIJI software (National Institutes of Health, Bethesda, MD). Relative area of PSR-positive signal was quantified above a threshold that was set based on the section with the most staining, as previously published [13, 19]. This threshold was kept constant for all images analyzed for each experiment and fold-change was calculated over young ovarian sections. To quantify relative ECM-Enriched area from Hematoxylin-stained tissue sections, thresholding was used to measure total ovarian area and area with residual nuclei was measured manually using the freehand tool in FIJI.

### Statistical analysis

Statistical analysis was performed using GraphPad Prism Software (La Jolla, CA, USA). *T*-tests were performed to evaluate differences between groups. *P* values < 0.05 were considered significant. The number of biological replicates (N) for each experiment is listed in the figure legends.

### Tissue lysis and homogenization

Ovarian tissue from reproductively young (*N* = 5; 5 native and 5 ECM-enriched ovaries) and reproductively old (*N* = 5; 5 native and 5 ECM-enriched ovaries) mice was prepared for proteomic analysis. Samples were homogenized in 600 μL lysis buffer containing 8 M urea, 2% sodium dodecyl sulfate (SDS), 1 μM trichostatin A (TSA), 3 mM nicotinamide adenine dinucleotide (NAD), 75 mM sodium chloride, and 1× protease and phosphatase inhibitor cocktail (Thermo Fisher Scientific, Waltham, MA, USA) in 200 mM triethylammonium bicarbonate (TEAB) by adding them to 2.0 mL safe-lock tubes (VWR International, Radnor, PA, USA) containing stainless steel beads and subjected to three intervals of high-speed shaking (25 Hz, 1 min) using a Qiagen TissueLyser II (Qiagen, Hilden, Germany). Tissue homogenates were centrifuged at 15,700 × g for 10 min at 4°C, and the supernatant was collected for label-free quantitative proteomics experiments. Protein concentration was determined using Bicinchoninic Acid (BCA) assay (Thermo Fisher Scientific, Waltham, MA, USA).

### Protein digestion and desalting

Aliquots of 200 μg protein lysates for each sample were brought to the same overall volume of 100 μL with water, reduced using 20 mM dithiothreitol in 50 mM TEAB at 50°C for 10 min, cooled to room temperature (RT) and held at RT for 10 min, and alkylated using 40 mM iodoacetamide in 50 mM TEAB at RT in the dark for 30 min. Samples were acidified with 12% phosphoric acid to obtain a final concentration of 1.2% phosphoric acid. S-Trap buffer consisting of 90% methanol in 100 mM TEAB at pH ~7.1, was added and samples were loaded onto the S-Trap micro spin columns (Protifi, Fairport, NY, USA). The entire sample volume was spun through the S-Trap micro spin columns at 4,000 × g at RT, binding the proteins to the micro spin columns. Subsequently, S-Trap micro spin columns were washed twice with S-Trap buffer at 4,000 × g at RT and placed into clean elution tubes. Samples were incubated for one-hour at 47^o^C with sequencing grade trypsin (Promega, San Luis Obispo, CA) dissolved in 50 mM TEAB at a 1:25 (w/w) enzyme:protein ratio, and then digested overnight at 37^o^C.

Peptides were sequentially eluted from S-Trap micro spin columns with 50 mM TEAB, 0.5% formic acid (FA) in water, and 50% acetonitrile (ACN) in 0.5% FA. After centrifugal evaporation, samples were resuspended in 0.2% FA in water and desalted with Oasis 10-mg Sorbent Cartridges (Waters, Milford, MA, USA). The desalted elutions were then subjected to an additional round of centrifugal evaporation and re-suspended in 0.1% FA in water at a final concentration of 1 μg/μL. Eight microliters of each sample was diluted with 2% ACN in 0.1% FA to obtain a concentration of 400 ng/μL. One microliter of indexed Retention Time Standard (iRT, Biognosys, Schlieren, Switzerland) was added to each sample, thus bringing up the total volume to 20 μL [88].

### Mass spectrometric analysis

Reverse-phase HPLC-MS/MS analyses were performed on a Dionex UltiMate 3000 system coupled online to an Orbitrap Exploris 480 mass spectrometer (Thermo Fisher Scientific, Bremen, Germany). The solvent system consisted of 2% ACN, 0.1% FA in water (solvent A) and 80% ACN, 0.1% FA in ACN (solvent B). Digested peptides (400 ng) were loaded onto an Acclaim PepMap 100 C_18_ trap column (0.1 × 20 mm, 5 μm particle size; Thermo Fisher Scientific) over 5 min at 5 μL/min with 100% solvent A. Peptides (400 ng) were eluted on an Acclaim PepMap 100 C_18_ analytical column (75 μm × 50 cm, 3 μm particle size; Thermo Fisher Scientific) at 300 nL/min using the following gradient: linear from 2.5% to 24.5% of solvent B in 125 min, linear from 24.5% to 39.2% of solvent B in 40 min, up to 98% of solvent B in 1 min, and back to 2.5% of solvent B in 1 min. The column was re-equilibrated for 30 min with 2.5% of solvent B, and the total gradient length was 210 min. Each sample was acquired in data-independent acquisition (DIA) mode [27–29]. Full MS spectra were collected at 120,000 resolution (Automatic Gain Control (AGC) target: 3e6 ions, maximum injection time: 60 ms, 350-1,650 *m/z*), and MS2 spectra at 30,000 resolution (AGC target: 3e6 ions, maximum injection time: Auto, Normalized Collision Energy (NCE): 30, fixed first mass 200 *m/z*). The isolation scheme consisted of 26 variable windows covering the 350-1,650 *m/z* range with an overlap of 1 *m/z* [28].

### DIA data processing and statistical analysis

DIA data were processed in Spectronaut (version 14.10.201222.47784) using directDIA. Data extraction parameters were set as dynamic and non-linear iRT calibration with precision iRT was selected. Data were searched against the *Mus musculus* reference proteome with 58,430 entries (UniProtKB-TrEMBL), accessed on 01/31/2018. Trypsin/P was set as the digestion enzyme and two missed cleavages were allowed. Cysteine carbamidomethylation was set as a fixed modification while methionine oxidation and protein N-terminus acetylation were set as dynamic modifications. Identification was performed using 1% precursor and protein *q*-value. Quantification was based on the peak areas of extracted ion chromatograms (XICs) of 3 – 6 MS2 fragment ions, specifically b- and y-ions, with local normalization and *q*-value sparse data filtering applied (Supplementary Tables 1, 2). In addition, iRT profiling was selected. Differential protein expression analysis comparing either (1) Young to Old Native or (2) Young to Old ECM-enriched was performed using a paired *t*-test, and *p*-values were corrected for multiple testing, using the Storey method [89]. Specifically, group wise testing corrections were applied to obtain *q*-values. Protein groups with at least two unique peptides, *q*-value < 0.01, and absolute Log_2_(fold-change) > 0.58 were considered significantly altered (Supplementary Figure 2, Supplementary Tables 3, 4).

### Statistical processing and pathway analysis

Partial least square-discriminant analysis (PLS-DA) of the proteomics data were performed using the package mixOmics in R (version 4.0.2; RStudio, version 1.3.1093) [90]. Volcano plots for differential analysis were generated using R (RStudio, version 1.3.1093). Annotation of matrisome proteins was performed using matrisome DB database [39, 40].

An over-representation analysis (ORA) was performed using Consensus Path DB-mouse (Release MM11, 14.10.2021), developed by the bioinformatics group at the Max Planck Institute for Molecular Genetics (Berlin, Germany) [91, 92]. The following comparisons were used to evaluate which gene ontology terms, including biological processes and molecular functions, were significantly enriched: (1) Old vs. Young Native and (2) Old vs. Young ECM-enriched. Gene ontology terms identified from the ORA were subjected to the following filters: *q*-value < 0.05, term category = b (biological processes) or m (molecular functions), and term level = 4 for biological processes or 3 for molecular functions. Dot plots were generated using the ggplot2 package in R (version 4.0.5; RStudio, version 1.4.1106) to visualize significantly enriched biological processes from each comparison [93].

### Data availability

Raw data and complete MS data sets have been uploaded to the Mass Spectrometry Interactive Virtual Environment (MassIVE) repository, developed by the Center for Computational Mass Spectrometry at the University of California San Diego, and can be downloaded using the following link: https://massive.ucsd.edu/ProteoSAFe/dataset.jsp?task=13fe684e74b34dcaa087c6960e5a40ac (MassIVE ID number: MSV000091790; ProteomeXchange ID: PDX041789).

## Supplementary Materials

Supplementary Figures

Supplementary Table 1

Supplementary Table 2

Supplementary Table 3

Supplementary Table 4

Supplementary Tables 5 and 6
