# Supplementary material for: Proteomic quantification of native and ECM-enriched mouse ovaries reveals an age-dependent fibro-inflammatory signature
Source: Aging (Albany NY). 2023 Oct 27;15(20):10821–55. doi: 10.18632/aging.205190 (PMC10637783; doi:10.18632/aging.205190)
Supplement: Supplementary Figures [file aging-15-205190-s001.pdf]

SUPPLEMENTARY FIGURES

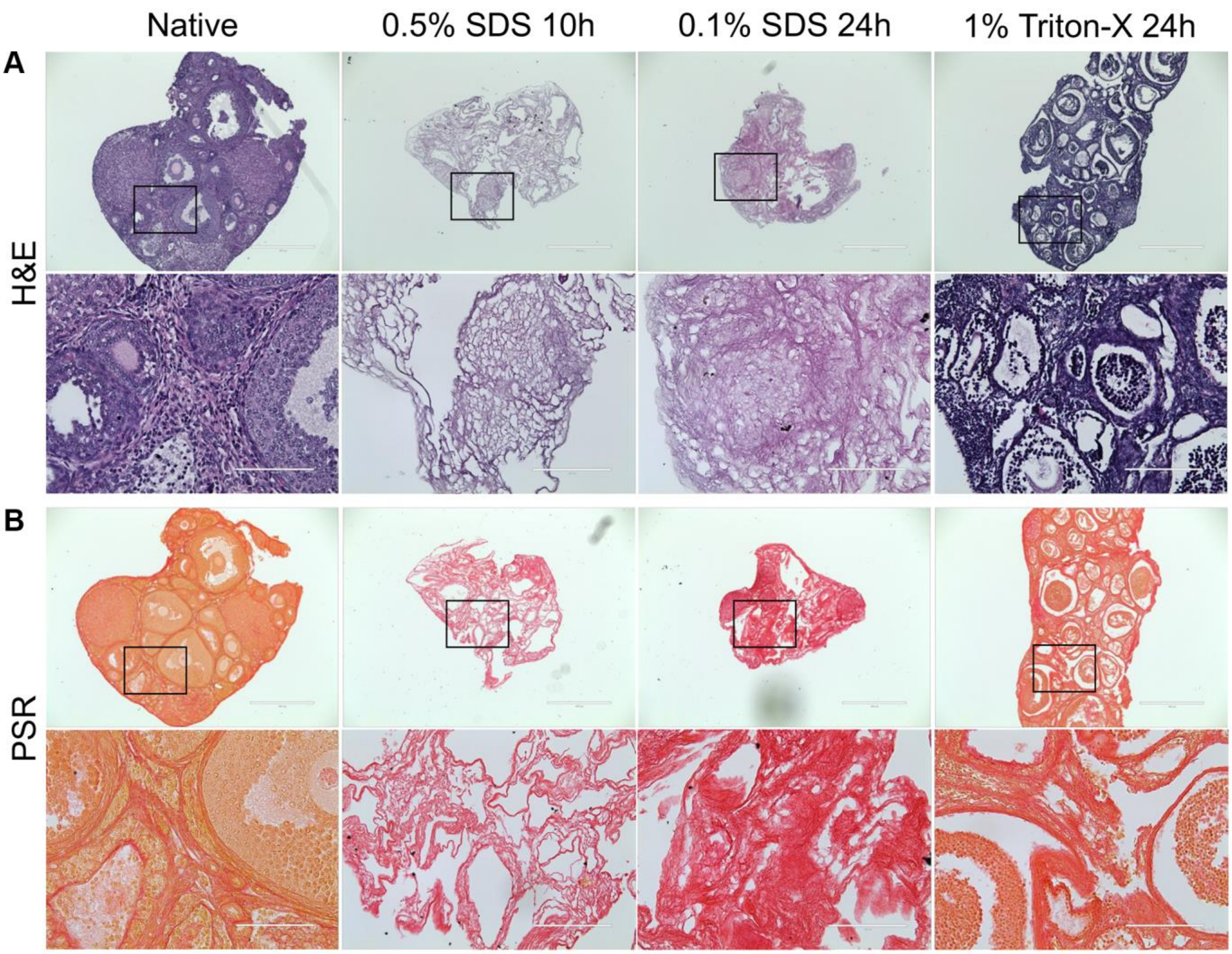

Supplementary Figure 1. Detergents used for ECM-enrichment. Representative images of H&E (**A**) and PSR (**B**) stained native ovarian tissue sections or ovarian tissue sections following treatment with 0.5% SDS for 10 h, 0.1% SDS for 24 h, or 1% Triton-X for 24 h. Bottom row of each panel is optical zoom of boxed region from top row. Scale bar for top row for each panel = 400 µm. Scale bar for bottom row for each panel = 100 µm. *N* = 1 ovary per group.

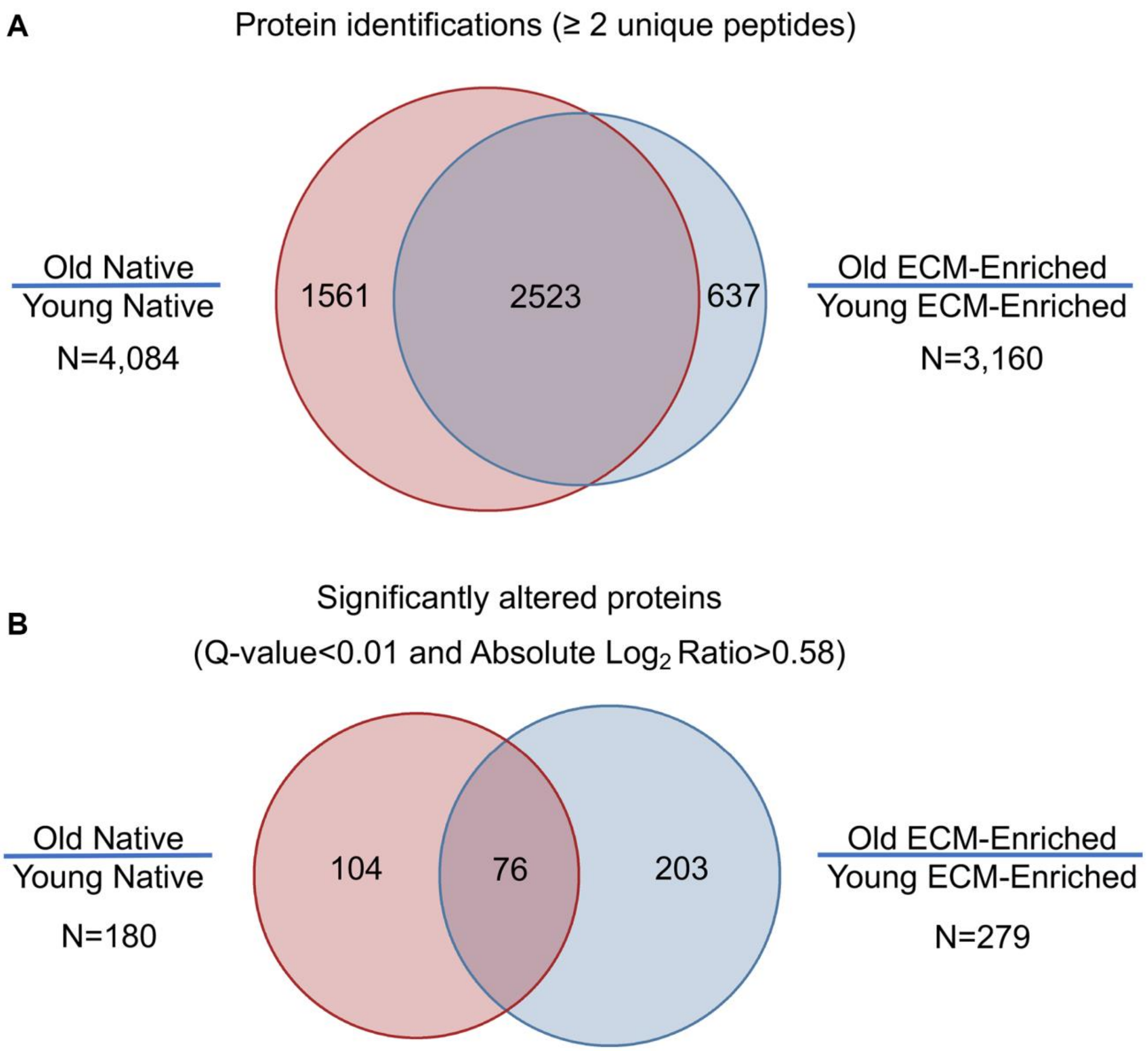

Supplementary Figure 2. The ovarian proteome is significantly altered during reproductive aging. (**A**) Comparison of protein identifications of native (*N* = 4,084) and ECM-enriched (*N* = 3,160) ovaries from reproductively young and old mice. Quantifiable proteins had 2 or more unique peptides per unique protein identification. (**B**) Comparison of proteins significantly altered with age in native (*N* = 180) and ECM-enriched (*N* = 279) ovaries. Criteria for significantly altered proteins are as follows: *q*-value < 0.01 and $|\log_2(\text{Old/Young})| > 0.58$.

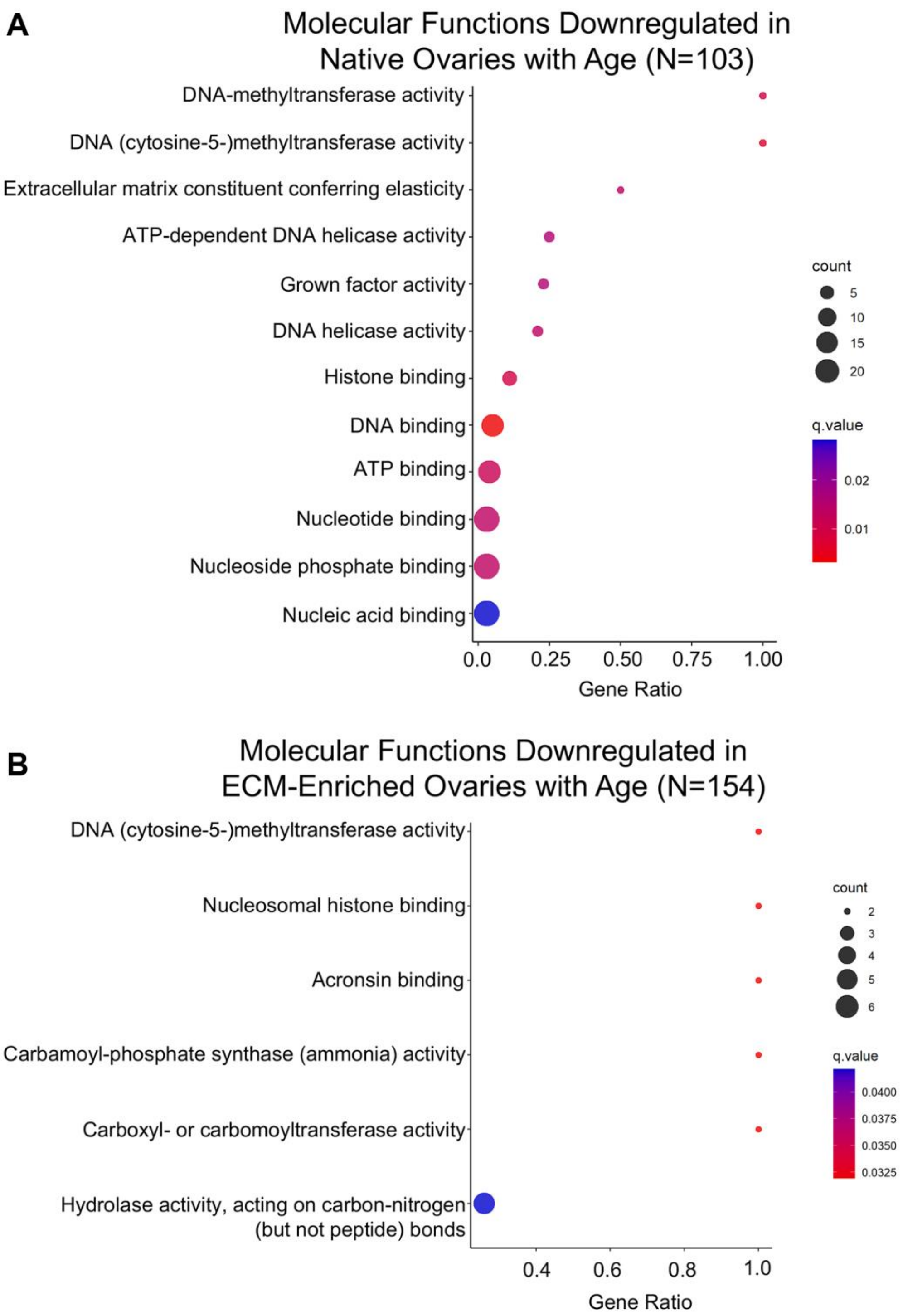

Supplementary Figure 3. Molecular functions associated with DNA replication, epigenetic modification, and DNA packaging are downregulated in the ovary with age. GO analysis of proteins significantly downregulated with age from (**A**) native and (**B**) ECM-enriched ovaries was performed using consensus pathway database (CPDB) at level 3, q-value < 0.05. The level of enrichment (corrected gene ratio) is depicted from 0–1, corresponding to 0–100% enrichment of the listed pathway.

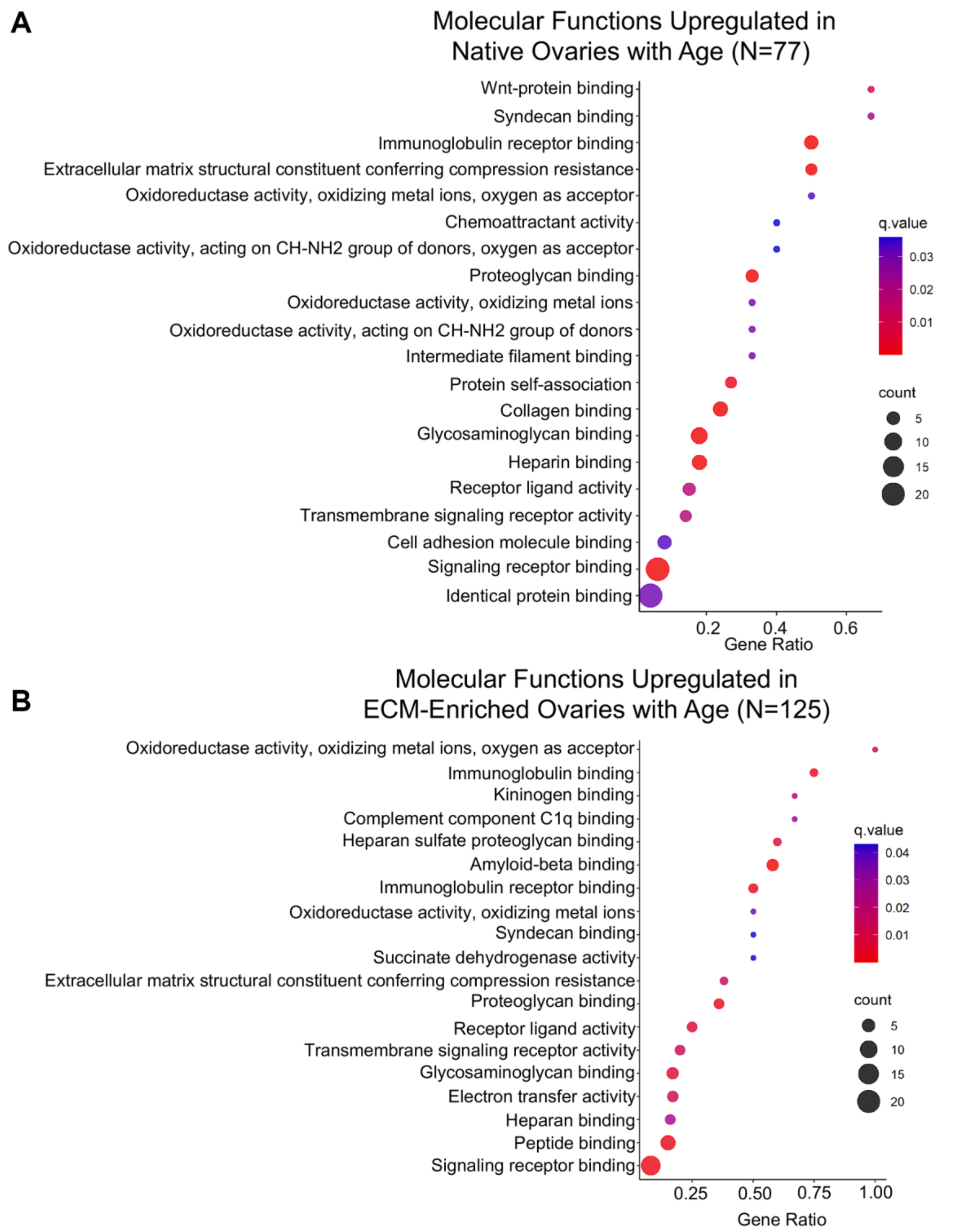

Supplementary Figure 4. Molecular functions associated with oxidative stress and ECM protein binding are upregulated in the ovary with age. GO analysis of proteins significantly upregulated with age from (**A**) native and (**B**) ECM-enriched ovaries was performed using consensus pathway database (CPDB) at level 3, q-value < 0.05. The level of enrichment (corrected gene ratio) is depicted from 0–1, corresponding to 0–100% enrichment of the listed pathway.
