# Supplementary material for: Proteomic quantification of native and ECM-enriched mouse ovaries reveals an age-dependent fibro-inflammatory signature
Source: Aging (Albany NY). 2023 Oct 27;15(20):10821–55. doi: 10.18632/aging.205190 (PMC10637783; doi:10.18632/aging.205190)
Supplement: Supplementary Tables 5 and 6 [file aging-15-205190-s006.pdf]

SUPPLEMENTARY TABLES

Supplementary Table 5. All matrisome proteins identified in native and ECM-enriched mouse ovaries.

Experimental group	Division	Category	Protein names
Native	Core Matrisome	ECM Glycoproteins	Fibulin-5, Elastin, Microfibril-associated glycoprotein 4, SPARC-related modular calcium-binding protein 1, SPARC-like protein 1, Adiponectin, Von Willebrand factor A domain-containing protein 9, Cysteine-rich with EGF-like domain protein 2, Multimerin-2, Cysteine-rich with EGF-like domain protein 1
	Matrisome-associated	ECM Regulators	Pregnancy zone protein, Cathepsin C, Murinoglobulin-2, Prothrombin, Kininogen-1, Inter-alpha-trypsin inhibitor heavy chain 1, Heparin cofactor 2, Cystatin-B, Cathepsin S, Prolyl 4-hydroxylase subunit alpha 1, Cystatin-C, Kininogen-2, Glia-derived nexin, Inter-alpha-trypsin inhibitor heavy chain 3, Alpha-1-antitrypsin 1-2, Carboxypeptidase N subunit 2, Pro-cathepsin H, Carboxypeptidase, Lysl oxidase homolog 2, Plasma protease C1 inhibitor, Corticosteroid-binding globulin, Angiotensinogen, Glycosaminoglycan xylosylkinase, Disintegrin and metalloproteinase domain-containing protein 17
		ECM-affiliated Proteins	Galectin, Chondroitin sulfate proteoglycan 4, Hemopexin, Plexin domain-containing protein 2, Galectin-related protein, Syndecan-1, Semaphorin-7a
		Secreted Factors	Anti-Mullerian hormone, S100 calcium-binding protein A1, Hepatocyte growth factor activator, Follistatin, Secreted frizzled-related protein 1
ECM-Enriched	Core Matrisome	Collagens	Collagen-type XI-α1 chain, Collagen-type V-α1 chain
		ECM Glycoproteins	Von Willebrand factor A domain-containing protein 1
	Matrisome-associated	ECM Regulators	Trypsin 10
		ECM-affiliated Proteins	Mannose-binding protein A, Complement component 1Q subunit B, C-type lectin domain family 10 member A
Both	Core matrisome	Collagens	Collagen-type V-α2 chain, Collagen-type III-α1 chain, Collagen-type IV-α1 chain, Collagen-type IV-α2 chain, Collagen-type VI-α6 chain, Collagen-type I-α2 chain, Collagen-type XV-α1 chain, Collagen-type I-α1 chain, Collagen-type XVIII-α1 chain, Collagen-type VI-α2 chain, Collagen-type VI-α1 chain, Collagen-type VI-α4 chain, Collagen-type VI-α4 chain, Collagen-type VI-α5 chain, Collagen-type XII-α1 chain, Collagen-type VI-α3 chain
		ECM Glycoproteins	Periostin, Growth arrest-specific protein 6, Microfibrillar-associated protein 2, SPARC, Peroxidasin, Latent-transforming growth factor beta-binding protein 3, EGF-containing fibulin-like extracellular matrix protein 2, Laminin gamma 3, Vitronectin, Procollagen C-endopeptidase enhancer 1, Matrilin-2, Latent-transforming growth factor beta-binding protein 4, Dermatopontin, Latent-transforming growth factor beta-binding protein 1, Extracellular matrix protein 1, Tubulointerstitial nephritis antigen-like, Insulin-like growth factor-binding protein 7, Fibulin-1, Thrombospondin-4, Lactadherin, Zona pellucida sperm-binding protein 1, Zona pellucida sperm-binding protein 3, Adipocyte enhancer-binding protein 1, Fibrinogen alpha chain, EGF-containing fibulin-like extracellular matrix protein 1, Fibrinogen gamma, Elastin microfibril interface-located protein 1, Fibrillin-2, Zona pellucida sperm-binding protein 2, Von Willebrand factor A domain-containing protein 5a, Fibrinogen beta chain, Laminin alpha 5, Transforming growth factor-beta-induced, Nidogen-2, Laminin alpha 4, Thrombospondin-1, Agrin, Laminin alpha 2, Nidogen-1, Laminin beta 1, Laminin beta 2, Laminin gamma 1, Fibronectin, Laminin alpha 1, Tenascin-X, Fibrillin-1, Tenascin
		Proteoglycans	Basement membrane-specific heparan sulfate proteoglycan core protein, Mimecan, Asporin, Lumican, Biglycan, Decorin, Prolargin
	Matrisome-associated	ECM Regulators	Alpha-2-antiplasmin, Alpha-1-microglobulin/bikunin precursor, N-glycanase 1, Coagulation factor IX, Disintegrin and metalloproteinase domain-containing protein 10, Cathepsin F, Coagulation factor X, Coagulation factor XVIII A chain, Antithrombin-III, Cathepsin Z, Cathepsin L, Disintegrin and metalloproteinase domain-containing protein 9, High-temperature requirement A serine peptidase 1, Astacin-like metalloendopeptidase, Serine protease inhibitor B6B, Serine protease

			inhibitor A3K, Serine protease inhibitor A3G, Histidine-rich glycoprotein, Cathepsin B, Cathepsin D, Serine protease inhibitor B9, Inter-alpha-trypsin inhibitor heavy chain 5, Leukocyte elastase inhibitor A, Serine protease inhibitor H1, Transglutaminase 2, Plasminogen
		ECM-affiliated Proteins	Plexin-A1, Glypican-4, Plexin-B1, Glypican-1, Collectin-12, Galectin-9, Mannose-binding protein C, Galectin-1, Galectin-3, Annexin A11, Lectin mannose-binding 1, Plexin-B2, Annexin A7, Annexin A3, Annexin A4, Annexin A5, Annexin A1, Annexin A2, Annexin A6
		Secreted Factors	Wnt-4, S100 calcium-binding protein A11, S100 calcium-binding protein A6, S100 calcium-binding protein A10, S100 calcium-binding protein A4, Inhibin alpha chain, Secreted frizzled-related protein 4, Host cell factor 1, S100 calcium-binding protein A16, S100 calcium-binding protein A16, Fibroblast growth factor 2

Supplementary Table 6. Matrisome proteins significantly altered with advanced age.

Change in expression with age	Experimental group	Division	Category	Protein names
Upregulated	Native	Core matrisome	Collagens	Collagen-type VI-α6 chain
			ECM Glycoproteins	Dermatopontin, Transforming growth factor-beta-induced
			Proteoglycans	Asporin, Biglycan
		Matrisome-associated	ECM Regulators	Cathepsin C, Cathepsin B, Cathepsin S, Heparin cofactor 2
			ECM-affiliated Proteins	Galectin-7
			Secreted Factors	Fibroblast growth factor 2, Secreted frizzled-related protein 1, Secreted frizzled-related protein 4, S100 calcium-binding protein A1
	ECM-Enriched	Core matrisome	ECM Glycoproteins	Fibrinogen alpha chain, Fibrinogen beta chain
			Proteoglycans	Mimecan
		Matrisome-associated	ECM Regulators	Cathepsin F, Cathepsin L, Coagulation factor IX, Histidine-rich glycoprotein
			ECM-affiliated Proteins	Glypican-1
			Secreted Factors	S100 calcium-binding protein A10, S100 calcium-binding protein A13, S100 calcium-binding protein A16
	Both	Core matrisome	ECM Glycoproteins	EGF-containing fibulin-like extracellular matrix protein 1, Matrilin-2, Periostin, Tenascin-C, Tenascin-X
			Proteoglycans	Decorin, Lumican
		Matrisome-associated	ECM Regulators	Alpha-1-microglobulin/bikunin precursor, Cathepsin D
			ECM-affiliated Proteins	Ficolin-1, Mannose-binding protein C
			Secreted Factors	Wnt-4
Downregulated	Native	Core matrisome	ECM Glycoproteins	Elastin, Fibulin-5
		Matrisome-associated	ECM Regulators	Glia-derived nexin
			Secreted Factors	Anti-Mullerian hormone
	ECM-Enriched	Core matrisome	Collagens	Collagen-type XI-α1 chain
			ECM Glycoproteins	Fibulin-1, Fibrillin-2, Thrombospondin-1, Zona pellucida sperm-binding protein 1, Zona pellucida sperm-binding protein 2, Zona pellucida sperm-binding protein 3
			Proteoglycans	Versican

		Matrisome-associated	ECM Regulators	Serpin H1, Trypsin 10
			ECM-affiliated Proteins	Annexin A3, Annexin A5
			Secreted Factors	S100 calcium-binding protein A4
	Both	Core matrisome	ECM Glycoproteins	Thrombospondin-4
		Matrisome-associated	ECM Regulators	Astacin-like metalloendopeptidase, Procollagen-lysine 2-oxoglutarate 5-dioxygenase
			Secreted Factors	Inhibin alpha chain
